# Adipose-Derived Mesenchymal Stromal Cells Treated with Interleukin 1 Beta Produced Chondro-Protective Vesicles Able to Fast Penetrate in Cartilage

**DOI:** 10.3390/cells10051180

**Published:** 2021-05-12

**Authors:** Alessandra Colombini, Enrico Ragni, Leonardo Mortati, Francesca Libonati, Carlotta Perucca Orfei, Marco Viganò, Marco Brayda-Bruno, Laura de Girolamo

**Affiliations:** 1 Laboratorio di Biotecnologie Applicate all’Ortopedia, IRCCS Istituto Ortopedico Galeazzi, I-20161 Milano, Italy; enrico.ragni@grupposandonato.it (E.R.); francesca.libonati@grupposandonato.it (F.L.); carlotta.perucca@grupposandonato.it (C.P.O.); marco.vigano@grupposandonato.it (M.V.); laura.degirolamo@grupposandonato.it (L.d.G.); 2 INRIM-Istituto Nazionale di Ricerca Metrologica, 10135 Torino, Italy; l.mortati@inrim.it; 3 III Spine Surgery-Scoliosis Department, IRCCS Istituto Ortopedico Galeazzi, 20161 Milano, Italy; marco.brayda@spinecaregroup.it

**Keywords:** adipose-derived mesenchymal stromal cells, extracellular vesicles, miRNA, interleukin 1 beta, cartilage, osteoarthritis, nonlinear optical microscopy, time-lapse

## Abstract

The study of the miRNA cargo embedded in extracellular vesicles (EVs) released from adipose-derived mesenchymal stromal cells (ASC) preconditioned with IL-1β, an inflammatory stimulus driving osteoarthritis (OA), along with EVs-cartilage dynamic interaction represent poorly explored fields and are the purpose of the present research. ASCs were isolated from subcutaneous adipose tissue and EVs collected by ultracentrifugation. Shuttled miRNAs were scored by high-throughput screening and analyzed through bioinformatics approach that predicted the potentially modulated OA-related pathways. Fluorescently labeled EVs incorporation into OA cartilage explants was followed in vitro by time-lapse coherent anti-Stokes Raman scattering; second harmonic generation and two-photon excited fluorescence. After IL-1β preconditioning, 7 miRNA were up-regulated, 4 down-regulated, 37 activated and 17 silenced. Bioinformatics allowed to identify miRNAs and target genes mainly involved in Wnt, Notch, TGFβ and Indian hedgehog (IHH) pathways, cartilage homeostasis, immune/inflammatory responses, cell senescence and autophagy. As well, ASC-EVs steadily diffuse in cartilage cells and matrix, reaching a plateau 16 h after administration. Overall, ASCs preconditioned with IL-1β allows secretion of EVs embedded with a chondro-protective miRNA cargo, able to fast penetrate in collagen-rich areas of cartilage with tissue saturation in a day. Further functional studies exploring the EVs dose-effects are needed to achieve clinical relevance.

## 1. Introduction

Osteoarthritis (OA) is a chronic pathology of the whole joint [1], leading to a progressive loss of bony, cartilaginous and synovial tissues homeostasis, driven and sustained by catabolic and inflammatory process.

Adipose-derived mesenchymal stromal cells (ASCs) based therapy has been used in some clinical trials to treat OA patients [2] as a tool potentially acting on several pathological targets given their immunomodulatory and trophic ability. In fact, ASCs respond in an adaptive way to inflammatory stimuli, in particular to interleukin-1β (IL-1β) [3], a central molecular regulator of the complex network of proteolytic enzymes, chemokines and cytokines leading the degenerative processes of OA [4]. In vitro, ASCs showed high responsiveness to IL-1β stimulation, resulting in the up-regulation of matrix metalloproteases (MMPs)-1, -3 and -13, angiogenic and mitogenic factors, cytokines controlling production, differentiation and function of macrophages, as well as molecules belonging to the insulin-like growth factor (IGF), platelet-derived growth factor (PDGF) and transforming growth factor (TGF) families and pro- (IL-1β and IL-8) and anti- (IL-1Ra) inflammatory cytokines [3].

In basal conditions, ASC released extracellular vesicles (EVs) with a preponderance of microRNAs (miRNAs) involved in OA cartilage and synovium protection (40%) over less than 10% of those involved in degenerative processes [5]. In addition, the EV cargo can be further modulated by preconditioning the mesenchymal stem cells (MSCs) with stimuli resembling the in vivo pathological environment [6,7,8]. ASCs inflammatory priming with interferon γ (IFNγ) determined a production of a 34.7% chondro-protective versus a 13.3% chondro-destructive miRNAs and a secretion of 26.2% versus 4.5% of M2- versus M1-macrophages-related miRNAs [9].

In addition to these evidences, a large number of miRNAs are able to modulate IL-1β induced extracellular cartilage matrix degradation and are involved in the control of the initiation and progression of OA [10,11,12]. For these reasons, the ASC preconditioning with IL-1β could represent an already unexplored strategy to produce functional EVs exploitable as cell-free therapeutic tool to induce whole joint homeostasis and prevent further OA progression.

In this specific context, it is essential to analyze also the EV interaction with the tissue and the cells resident therein to better understand their real therapeutic potential. Recently, the ability of ASC-EVs to penetrate in chondrocyte micromasses and cartilage explants from osteoarthritic patients was shown through a novel, real-time, microscopy technique [13].

The aim of the present study was to characterize the EVs obtained from ASCs treated with IL-1β in term of shuttled miRNAs and their kinetic of penetration in OA cartilage to understand the potential of this cell-free approach for treatment of OA.

## 2. Materials and Methods

### 2.1. ASCs Isolation, Expansion and Cartilage Collection

The study was approved by the local Institutional Review Board (M-SPER-015) and tissue samples were collected after obtaining patients’ informed written consent.

Subcutaneous adipose tissue from local hip fat deposit of three patients who underwent total hip replacement (2 females 53 and 56 y/o and 1 male 41 y/o) was collected and ASCs isolated by enzymatic digestion (37 °C, 30 min with 0.075% *w/v* type I collagenase (Worthington Biochemical, Lakewood, NJ, USA) [14].

The cells were cultured in minimum essential medium (αMEM), supplemented with 10% fetal bovine serum (FBS) (Lonza, Basel, Switzerland), 0.29 mg/mL L-glutamine, 100 U/mL penicillin, 100 μg/mL streptomycin, 10 mM 4-(2-hydroxyethyl)piperazine-1-ethanesulfonic acid (HEPES), 1 mM sodium pyruvate (all reagents from Thermo Fisher Scientific, Waltham, Massachusetts, USA) and 5 ng/mL fibroblast growth factor 2 (PeproTech, Rocky Hill, NJ, USA) at 37 °C, 5% CO_2_, and 95% humidity, expanded until passage 3. and characterized as previously reported [3,15].

Articular cartilage slices (100–250 μm × 3–5 mm length) from non-weight bearing areas of the femoral heads/necks of other two females and one male affected by OA (Kellgren Lawrence III–IV, 38–54 y/o) undergoing total hip arthroplasty were collected and used for kinetic tests performed through a real-time, microscopy technique within two day.

### 2.2. EVs Isolation

The ASCs at 90% confluency were primed with 1 ng/mL of IL-1β for 48 h following a standardized protocol [3]. After this treatment, the cells were washed three times with PBS before adding serum free medium for 48 h. The culture supernatants (30 mL) were collected and differentially centrifuged at 376× *g*—15′ for debris and floating cells removal, 1000× *g*—15′, 2000× *g*—15′ and twice at 4000× *g*—15′ each at 4 °C to obtain cleared products. The cleared supernatants were ultracentrifuged at 100,000× *g*—3 h, 4 °C in a 70Ti rotor (Beckman Coulter, Brea, California, CA, USA) to obtain EVs. EV pellets were suspended in 100 μL phosphate-buffered saline (PBS), counted and characterized as previously reported [15].

### 2.3. EV-Embedded miRNA Expression

The EV pellets were dissolved by Trizol and ribonucleic acid (RNA) was obtained by using miRNeasy Kit and RNeasy CleanUp Kit (Qiagen, Hilden, Germany). Synthetic ath-miR-159a as a spike-in were added during the extraction to each sample to monitor the technical variability during the processes.

Reverse transcription and preamplification were performed to prepare complementary desoxyribonucleic acids (cDNAs) used for real-time polymerase chain reaction (PCR) with the QuantStudio™ 12 K Flex OpenArray^®^ Platform (QS12Kflex, Thermo Fisher Scientific). The Open Array covered 754 human miRNA sequences from the Sanger miRBase v21 Gene, divided into A and B panels.

### 2.4. miRNA Data Normalization

The miRNA expression was analyzed by Expression Suite Software (Life Technologies). The spike-in was used to equalize A and B panels of the Open Array and for the equalization of technical differences during the whole process [16]. CRT of 27 was considered as a threshold for the presence/absence of amplification. Global mean, calculated from miRNAs amplified in all samples, was the normalization method [17]. The relative quantification 2−ΔCRT was used to determine the miRNA expression, shown as mean values ± SD.

### 2.5. miRNA Differential Expression Analysis

Paired t-test was applied to normalized CRT (Table S1) to identify miRNAs up- or down-regulated in IL-1β treated respect to untreated ASCs. At the same time, miRNAs with CRT above the set threshold only in IL-1β treated or only in untreated ASCs were considered as activated or silenced, respectively.

### 2.6. miRNA Target Prediction Analysis

The bioinformatics Mientournet tool (http://userver.bio.uniroma1.it/apps/mienturnet/, accessed on 1 February 2021) [18] was used for validated target prediction analysis, considering miRTarBase database for experimentally validated miRNA-target interactions and miRNAs up- or down-regulated and activated or silenced by IL-1β treatment separately.

As first step, miRNA-target enrichment was performed by setting as threshold at least two gene-miRNA interactions and the lists of miRNAs with common targets and of genes targeted by the same miRNAs were retrieved (Supplementary Tables S2–S5). Functional enrichment analysis was conducted by exploiting three different bioinformatics open-source tools: KEGG pathway, Reactome and Wikipathway, allowing for visualization and interpretation of pathways to support basic research. Significant pathways overrepresented within the targets of selected miRNAs are listed and represented by circles, starting with those that are common to at least two miRNAs. Circles are colored according to the significance of the enrichment and their size is proportional to the number of involved targets.

Ingenuity Pathway Analysis (IPA; Ingenuity^®^ Systems, Qiagen, Germany, www.ingenuity.com, accessed on 1 February 2021) was used to identify the experimentally observed miRNA involved in OA, by using the miRNA Target Filter tool.

### 2.7. Time-Lapse Microscopy for EVs Incorporation in Cartilage

The EV-cartilage explants co-cultures were performed by using 25 × 10^8^ carboxyfluorescein succinimidyl ester (CFSE)-labeled EVs per μL. The samples were followed over a period of 40 h. The imaged cartilage portion had 279.7 µm (405 pixels) width, 350.8 µm (508 pixels) height, a total depth of 210.5 µm (20 Z slices, 3 images per slice) and was measured each 15 min (0.25 h) for a total of 65 timeframes (16 h).

The multimodal microscope allowed to collect images with Coherent Anti-Stokes Raman Scattering (CARS) for the cellular lipidic structures combined with Second Harmonic Generation (SHG) techniques for the collagen of the extracellular matrix [19] and Two-Photon Excitation Fluorescence (TPEF) for the EVs [13].

In the microscopy setup CARS signal was targeted at the CH2 stretching mode (2848 cm^−1^), using a Nd:YVO4 laser emitting 10 ps pulses centered at 1064 nm (Picotrain, HighQLaser, Austria) as the Stokes field and the signal output of an Optical Parametric Oscillator (Levante Emerald OPO) (APE, Berlin, Germany) tuned at about 817 nm with a pulse width of 6 ps as the pump field. The laser repetition rate was 76 MHz. The two beams entered into an upright microscope (BX51WI, Olympus, Tokyo, Japan) through the scanning unit (FluoView FV300, Olympus, Tokyo, Japan). The same OPO signal output at 817 nm was used as excitation source of the TPEF and SHG processes. The total average power at the sample of the excitation pulses was set to about 40 mW. The excitation beams were focused on living samples using a water immersion objective (LUMPLFLN 40XW NA = 0.8, W.D. = 3.3 mm, Olympus) that was cleaned and sterilized with a solution 70% ethanol in water (v/v) before the experiment. A condenser objective (UPLSAPO 10× objective NA = 0.4, Olympus) was used to collect forward CARS signal at about 663 nm and SHG signal at about 408 nm that were optically filtered and then detected using two separate PMTs (R3896, Hamamatsu, Japan). The TPEF signal was acquired in a back-scattering geometry (epi-detection).

A microscope stage incubator (Okolab, Napoli, Italy) maintained 37 °C, 5% CO_2_ and the humidity range between 50 and 95% through an Active Humidity Controller.

### 2.8. Multimodal Microscopy Data Analysis

The depth of EV penetration during the time-lapse together with the average occupied area and volume were computed using a custom-made ImageJ plugins processing the 3D images as already described [13].

## 3. Results

The ASCs were characterized in two previously published works by our group, showing the expression of characteristic MSC cell-surface antigens, the lack of expression of emato-endothelial markers, their colony forming unit ability and multi-differentiation potential [3,15].

### 3.1. IL-1β Preconditioning Modulated the Expression of a Small Amount of miRNAs

Among all the 754 analyzed miRNAs, 417 miRNA were detected in either basal or primed condition (Table S1). Seven (miR-125a-3p, miR-134-5p, miR-222-5p, miR-146a-5p, miR-155-5p, miR-196b-5p and miR-520c-3p) were significantly up-regulated, in particular miR-146a-5p and miR-520c-3p that showed a Fc over 30, and 4 (miR-191-3p, miR-500a-5p, miR-656-3p and miR-1265) were significantly down-regulated by the IL-1β treatment (Table 1). Moreover, 37 miRNA were activated and 17 were silenced by the IL-1β stimulus (Table 2).

### 3.2. IL-1β Up-Regulated/Activated miRNAs Showed Interaction with Pathways and Process Relevant to OA

Among the IL-1β up-regulated miRNA, miR-155-5p followed by miR-146a-5p, miR-196b-5p, miR-125a-3p, miR-134-5p and miR-520c-3p showed the highest number of interactions (Table S2).

**Figure 1:**
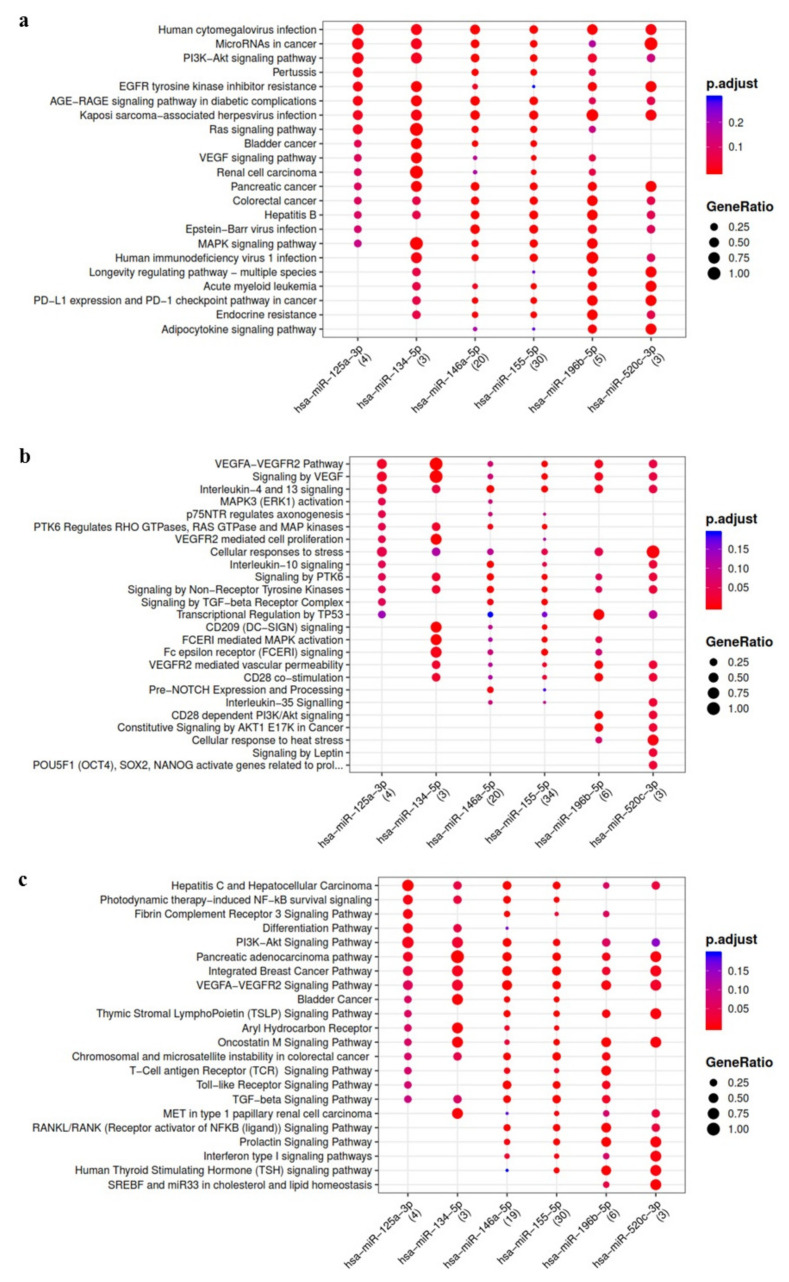
Functional enrichment analysis on miRNA up-regulated in ASC-EVs primed with IL-1β. The analysis was conducted by KEGG pathway (**a**) Reactome (**b**) and Wikipathway (**c**). Significant pathways are listed and represented by circles colored according to the significance of the enrichment and their size is proportional to the number of target genes regulating the described signaling pathways.

Bioinformatics analysis evidenced some relevant pathways modulated by IL-1β up-regulated miRNA and potentially involved in OA such as Wingless and Int-1 (Wnt), transforming growth factor beta (TGFβ), vascular endothelial growth factor (VEGF), interleukin-10 (IL-10), type I interferon (IFN1), Hippo and the other pathways related to proliferation, cell stress and lymphocyte mediated immunity (Figure 1).

**Figure 2:**
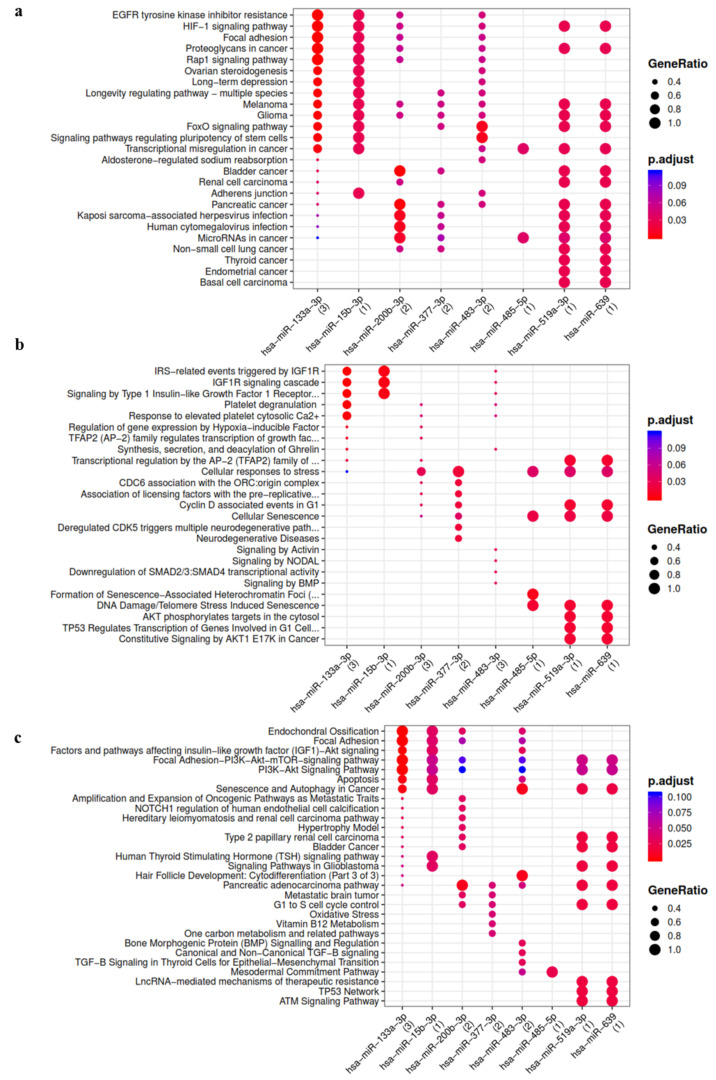
Functional enrichment analysis on miRNA activated in ASC-EVs primed with IL-1β. The analysis was conducted by KEGG pathway (**a**) Reactome (**b**) and Wikipathway (**c**). Significant pathways are listed and represented by circles colored according to the significance of the enrichment and their size is proportional to the number of target genes regulating the described signaling pathways.

Among the miRNAs activated by the treatment with IL-1β, miR-133a-3p, miR-483-3p, miR-200b-3p, miR-377-3p, miR-15b-3p, miR-485-5p and miR-519a-3p showed the highest number of interactions (Table S3). These IL-1β activated miRNA putatively modulate hypoxia-inducible factor 1 (HIF1), TGFβ, insulin like growth factor 1 (IGF-1), bone morphogenetic protein (BMP), Notch1, cell cycle, senescence, oxidative stress and endochondral ossification pathways (Figure 2).

On the contrary, IL-1β down-regulated miRNA (Table S4) showed no functional enrichment results.

**Figure 3:**
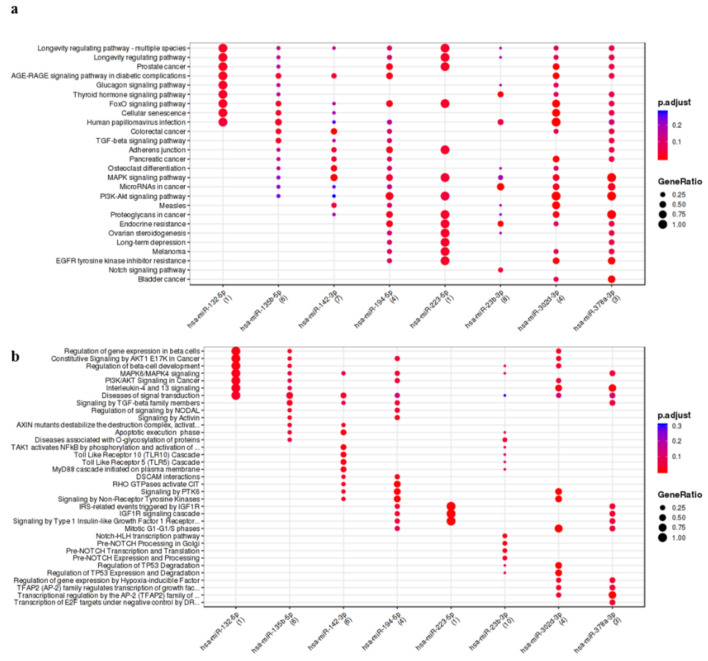
Functional enrichment analysis on miRNA silenced in ASC-EVs primed with IL-1β. The analysis was conducted by KEGG pathway (**a**) and Wikipathway (**b**). Significant pathways are listed and represented by circles colored according to the significance of the enrichment and their size is proportional to the number of target genes regulating the described signaling pathways.

Finally, concerning miRNA expressed in ASCs and silenced by inflammatory stimulus, miR-23b-3p followed by miR-142-3p, miR-135b-5p, miR-194-5p, miR-302d-3p, miR-378a-3p and miR-223-5p showed the higher number of interactions (Table S5). Notch, TGFβ and senescence are the pathways modulated by these miRNAs (Figure 3).

### 3.3. OA Focused Analysis Revealed Few miRNAs with a Key Role in the Wnt Pathway and Inflammatory Response

A further analysis focused on miRNA experimentally observed as involved in OA and modulated by IL-1β treatment allowed to identify 2 up-regulated, 4 activated and 4 silenced miRNAs (Table 3) and target genes mainly involved in Wnt pathway, in the regulation of immune and inflammatory responses, in Notch, TGFβ and Indian hedgehog (IHH) signaling, in cartilage homeostasis and in cell senescence and autophagy.

### 3.4. ASC-EVs Diffuse in Both Cells and Surrounding Matrix with Differential Kinetics

**Figure 4:**
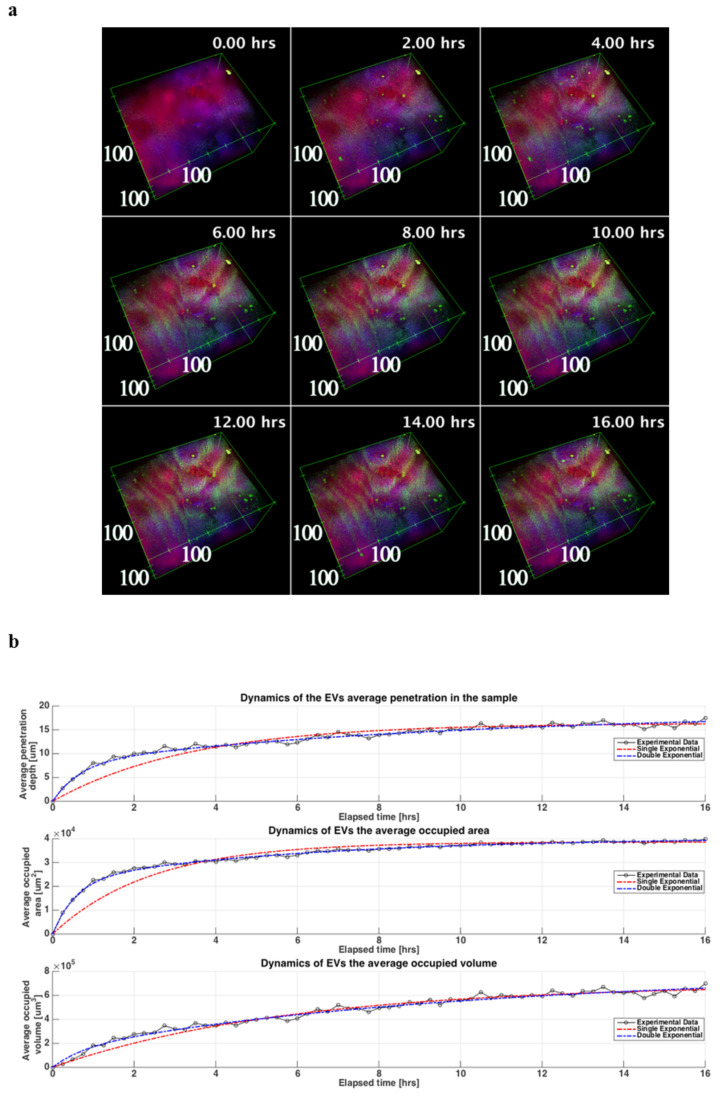
ASC-EVs kinetic of penetration in OA cartilage. (**a**) 3D reconstructions of the cartilage explant during the time-lapse experiment where the cells and the rich lipidic structures are shown in red (CARS signal at 2848 cm^−1^ from lipid structures), the collagen rich ECM is shown in blue (SHG signal from collagen) end the EVs are shown in green (TPEF signal from EVs fluorescence stain). In the box is shown the scale in μm, while in the upper right corner is depicted the corresponding time of the image during the experiment. The reconstructions are obtained from the acquired images and processed using 3Dviewer with ImageJ. (**b**) Three graphs of the dynamics of the EVs in the tissue during the time-lapse related to the depth, the area and the volume are showed. For each curve are also plotted the related fitting in blue the double exponential one (Equation (1) in Table S6) and in red the single exponential one (Equation (2) in Table S6).

The 3D reconstructions of the sample showed that ASC-EVs penetrated the cartilage explant along the observation time (Figure 4a).

The TPEF signal from the EVs was visible in the tissue already from the early steps of the experiment and further increased for both intensity and spatial distribution during the time-lapse up to 16 h when the signal reached a plateau.

Average penetration depth, area and volume for each time step showed a transient dynamic that can be better fitted using a function that is a sum of two exponential transients (Equation (1) in Table S6), with respect a single exponential transient (Equation (2) in Table S6) as it can be seen also in Figure 4b and from the sum of the squared errors (SSE) parameter that it is lower when Equation (1) is used. Therefore, two distinct kinetics, one faster than the other, differing by one order of magnitude were observed.

**Figure 5:**
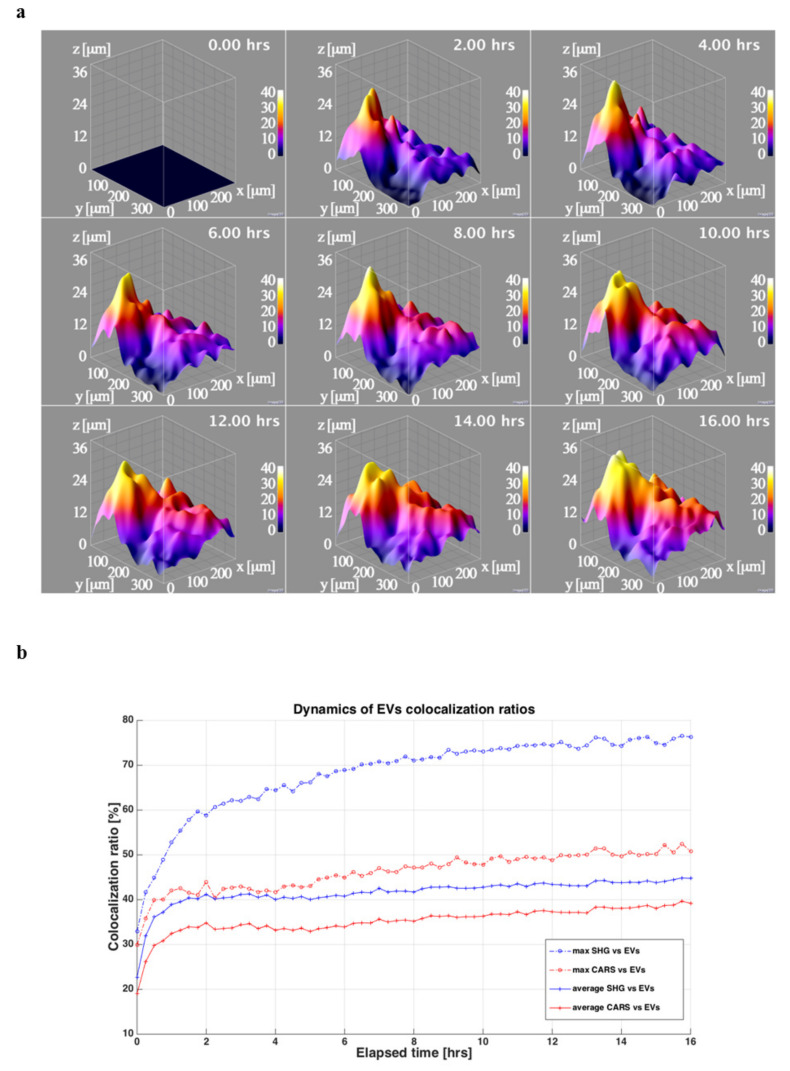
ASC-EVs diffusion in OA cartilage cells and matrix. (**a**) 3D surfaces showing the EVs penetration depths extracted from the acquired 3D imaging. In the upper corner are visible the corresponding time in the time-lapse experiment. The 3D surface graphs are obtained from the extracted EVs penetration depths images using the ImageJ3D plugin setting smoothing value at 10 pixels. (**b**) Dynamics of the co-localization ratios between the cells and the lipidic structure (CARS) and the EVs in red and between the collagen (SHG) in the ECM and the EVs in blue. For each ratio is reported the average (continuous line) and the maximum value (dash-dotted line) in the Z stack for that time step.

EVs penetration showed a shape depending on the morphology of the cartilage, reaching locally about 40 μm of depth at the end of experiment (Figure 5a), although there was a comprehensive rise on the penetration of the EVs along the whole sample.

The EVs were present in both the cells (CARS signal from lipid structures) and the extracellular matrix (ECM) (SHG signal from collagen) with a highest prevalence in the latter. This effect could be also observed analyzing the co-localization ratios of the EVs with respect to CARS signal and to SHG signal (Figure 5b). Even the co-localization ratios increased with the time during the experiment with a faster pace in the initial steps of the time-lapse and a gradually slowing down towards the plateau.

## 4. Discussion

The main findings of the present study are the OA-cartilage protective potential of the EV-embedded miRNAs released from adipose-derived mesenchymal stem cells subjected to preconditioning with IL-1β, together with their ability to quickly penetrate into cartilage.

Of note to envision clinical and off-the-shelf use of ASC-EVs, agreement was observed between the results obtained in the present work using ASCs from local hip fat and those previously published, with ASCs obtained from abdomen liposuction [5]. In fact, the list of the 100 more expressed EV-embedded miRNA in basal condition identified in the present study showed a 78% of matching with the previously published data of our research group [5], which in turn showed a 71–73% of agreement with another recently published dataset [20]. These results reinforce the concept that ASCs contain EVs with a stable miRNA content, regardless of the site of adipose tissue harvest, with a consistent reproducibility among different donors, even with respect to different ages.

As previously observed following IFNγ stimulation [9], the treatment with IL-1β does not induce strong changes in the cargo of the ASC-EVs in terms of inflammatory regulators, but it prompts the ASCs to up-regulate or produce miRNAs with chondro-protective potential, rather than to down-regulate or silence miRNAs with pro-inflammatory features.

In particular, miR-146a-5p and miR-520c-3p showed the highest and similar up-regulation (about 32–33 Fc) after priming with IL-1β. miR-146a was reported as related to OA pathogenesis and prevalence [21], according to OA severity. In fact, it is particularly expressed in low-grade OA cartilage and inversely correlated with cartilage degeneration in late-stage OA cartilage [22,23,24,25]. The expression of miR-146a-5p is induced by pro-inflammatory stimuli, particularly IL-1β, and it is involved in the regulation of both catabolic and anabolic processes driving cartilage homeostasis, neo-angiogenesis, and inflammatory functions in cartilage and synovium [24,26,27]. At experimental level, miR-146a is highly upregulated in IL-1β treated chondrocytes, in surgically-induced osteoarthritis in animals, and in response to mechanical stress [28,29]. Furthermore, miR-146a directly decreases Smad4 expression in chondrocytes, thereby disrupting signal transduction of TGFβ, resulting in an increase of VEGF and reduced cellularity in cartilage tissue [28].

The other highly up-regulated miRNA, miR-520c-3p, was found to be involved in the promotion of Wnt signaling, in the regulation of the inflammatory response and of the chondrogenic process [30].

Among other miRNAs of interest, miR-155-5p interacts with the greatest number of up-regulated or activated genes and it is supposed to be involved in aging, oxidative stress, modulation of Wnt pathway and inflammatory response. Notably, over expression of miR-155-5p and miR-146a was observed in synovial tissue and synovial fibroblasts derived from patients with rheumatoid arthritis in comparison to OA samples [31]. Moreover, the expression of these miRNA is up-regulated in synovial fibroblasts following stimulation with pro inflammatory mediators and its over expression indirectly counteracts the MMP-1 and -3 induction by pro-inflammatory cytokines, suggesting a protective role in arthritic joints [31,32].

Similar to what observed for the up-regulated miRNAs, some activated by IL-1β appeared as potentially involved in cartilage homeostasis such as miR-377-3p, miR-127-5p and miR-483-3p [33]. In particular, the latter was down-regulated in hypertrophic chondrocytes [34] but it was up-regulated in human and mouse OA tissues, suggesting a reparative role of this miRNA achieved through the promotion of an anabolic response and the inhibition of hypertrophic differentiation [10,33,35,36]. miR-127-5p, also playing a central role in cartilage homeostasis, was significantly down-regulated by IL-1β in human OA chondrocytes [37] and behaved as a suppressor of MMP-13 and IL-1β-mediated catabolic effects in chondrocytes [38].

Among the miRNAs silenced by IL-1β priming, miR-23b-and miR-378a-5p have a relevant role in OA, with the former being involved in chondrocyte differentiation and cartilage homeostasis [33,39], and the latter considered as a marker of late-stage OA [40].

Overall, several signaling pathways such as Wnt, TGFβ, Hippo, Notch and IHH, representing promising target for OA therapy [41], are stimulated by IL-1β in ASCs and shared with the ones activated by chondrocytes in presence of the same inflammatory stimulus. As a consequence, in chondrocytes there are a plethora of chondro-destructive processes and molecules activated by the cross talk of IL-1β with these pathways [4] that promote alteration of cartilage homeostasis, anabolic processes [42,43,44,45] and hypertrophic differentiation of chondrocytes [46] and that could be targeted by the miRNAs observed in IL-1β stimulated ASCs.

To result in a clinically effective product, the chondro-protective miRNA cargo of the EVs derived from IL-1β-primed ASCs must reach the target cells, crossing a complex matrix such as that of cartilaginous tissue. In this regard, it has been observed that the EVs derived from ASCs have the ability to fast penetrate for several micrometers into the cartilage, arriving predominantly at the matrix, but also reaching the cells. Herein presented results confirmed the quick EVs penetration along 30–40 μm depth already observed after a 5 h time-lapse in both micromasses and cartilage explants with the same technology [13], and further extended both intensity and spatial distribution measurements up to 16 h. Moreover, the observation of two diffusion kinetics of EVs suggests an active and steady initial process, followed by a more passive and slower one, which could be linked both to the differences in EVs penetration in the collagen-rich areas, but also to the progressive achievement of saturation, considering the 16 h as the identified time point when the signal reached a plateau. Finally, the highest EVs prevalence in ECM than in cells observed in experiments conducted on cartilage explants was not previously observed in micromasses [13], where a more homogenous EVs diffusion was reported. These data evidencing differences in EVs kinetics between the two 3D in vitro models might be due to the different cell to matrix ratios and local homogeneity, both higher in micromasses, and suggest the use of cartilage specimens for kinetic studies being more representative of the real cartilage physiology. Starting from these results, further functional studies, through potency and distribution assays, related to the EVs dose-effects will help to better define the optimal dosage and administration frequency to achieve a relevant clinical efficacy for the OA treatment.

## 5. Conclusions

Extracellular vesicles obtained from ASCs isolated from subcutaneous adipose tissue and preconditioned with IL-1β have a chondro-protective miRNA content and are able to fast penetrate cartilage, mainly in collagen-rich areas, likely reaching a tissue saturation after 16 h of administration.

## Figures and Tables

**Table 1:** Up- or down-regulated miRNAs by the IL-1β treatment. Fc= fold change.

Up-Regulated			Down-Regulated		
miRNA	Fc	*p*-Value	miRNA	Fc	*p*-Value
miR-125a-3p	2.2	0.04	miR-191-3p	0.4	0.02
miR-134-5p	5.7	0.007	miR-500a-5p	0.5	0.05
miR-222-5p	3.0	0.02	miR-656-3p	0.3	0.01
miR-146a-5p	33.2	0.004	miR-1265	0.5	0.04
miR-155-5p	4.6	0.02			
miR-196b-5p	2.4	0.003			
miR-520c-3p	32.2	0.05			

**Table 2:** Activated or silenced miRNAs by the IL-1β treatment.

Activated	Silenced
let-7f-2-3p	hsa-let-7f-1-3p
let-7i-3p	miR-132-5p
miR-100-3p	miR-122-3p
miR-10a-3p	miR-130b-5p
miR-127-5p	miR-135b-5p
miR-1282	miR-142-3p
miR-1290	miR-187-3p
miR-1304-5p	miR-194-5p
miR-1324	miR-223-5p
miR-133a	miR-23b-3p
miR-15b-3p	miR-302d-3p
miR-181c-3p	miR-378a-3p
miR-200b-3p	miR-518d-3p
miR-29b-2-5p	miR-566
miR-326	miR-589-3p
miR-361-3p	miR-623
miR-376b	miR-628-3p
miR-377-3p	
miR-380-3p	
miR-431-5p	
miR-449b-5p	
miR-483-3p	
miR-485-5p	
miR-489	
miR-511	
miR-517a-3p; miR-517b-3p	
miR-519a-3p	
miR-520b	
miR-523-3p	
miR-541-3p	
miR-550a-5p	
miR-551b-5p	
miR-604	
miR-639	
miR-646	
miR-7-2-3p	
miR-92a-1-5p	

**Table 3:** miRNA experimentally observed as involved in osteoarthritis and modulated by IL-1β treatment.

	miRNA	Target Genes	miRNA Properties
**Up-regulated**	miR-146a-5p	*SMAD4, VEGF, Bcl2*	TGFβ signaling inhibition, modulate chondrocytes apoptosis/autophagy and inflammatory functions
	miR-155-5p	*CEBPB*, *CTNNB1*, *SMAD1*, *TCF7L2*	Chondroprotective (suppress MMP-1 and MMP-3 production), Wnt signaling inhibition, modulate inflammatory functions
	miR-520c-3p	*DKK1*, *RELA, SEMA3C*	Wnt signaling activation, modulate inflammatory functions and chondrogenesis
**Activated**	miR-127-5p	*MMP13*	Chondroprotective (suppress MMP-13 production)
	miR-326	*SMO*	Chondroprotective
	miR-377-3p	*CART1*	Cartilage homeostasis
	miR-449b-5p	*JAG1, NOTCH1, SIRT1*	Chondroprotective (NOTHC signaling inhibition), senescence dysregulation
	miR-483-3p	*ACAN*	Cartilage homeostasis
	miR-519a-3p	*PRKAA1*	Senescence and autophagy dysregulation
	miR-520b	*DKK, RELA*	Wnt signaling activation, modulate inflammatory functions
**Silenced**	miR-135b-5p	*RUNX2, SMAD5*	Wnt signaling inhibition, inhibition of osteogenic differentiation
	miR-23b-3p	*HES1, NOTCH1, PRKACB, CRTAP*	Chondroprotective (NOTHC signaling inhibition), cartilage homeostasis
	miR-302d-3p	*DKK1, RELA*	Wnt signaling activation, modulate inflammatory functions
	miR-378a-3p	*CASP9*	Marker of late stage OA

## Data Availability

The datasets generated during and/or analysed during the current study are available in the OSF repository: https://osf.io/zde6p/?view_only=4a627ce15e3c42c4a926e3194cdb59d9, accessed on 1 February 2021.

## Supplementary Materials

The following are available online at https://www.mdpi.com/article/10.3390/cells10051180/s1, Table S1: normalized CRT values for detected EV-embedded miRNAs, Table S2: target genes of miRNAs up-regulated in ASCs treated with IL-1β, Table S3: target genes of miRNAs activated in ASCs treated with IL-1β, Table S4: target genes of miRNAs down-regulated in ASCs treated with IL-1β, Table S5: target genes of miRNAs silenced in ASCs treated with IL-1β, Table S6: fitting parameters related to the EVs average depth, occupied area and occupied volume are listed. The Equation (1) corresponds to the double exponential function used to fit the experimental data, while the Equation (2) corresponds to the single exponential function. For each fitting equation are showed the computed parameters and the Sum of the Squared Errors (SSE) and the coefficient of determination (R2).
