# Supplementary material for: Adipose-Derived Mesenchymal Stromal Cells Treated with Interleukin 1 Beta Produced Chondro-Protective Vesicles Able to Fast Penetrate in Cartilage
Source: Cells. 2021 May 12;10(5):1180. doi: 10.3390/cells10051180 (PMC8151616; doi:10.3390/cells10051180)
Supplement: Supplementary file 1 [file cells-10-01180-s001.zip › Table S1.pdf]

Table S1: normalized C_{RT} values for detected EV-embedded miRNAs.

**miRNA**	**ASC1**	**ASC2**	**ASC3**	**ASC1+IL-1β**	**ASC2+IL-1β**	**ASC3+IL-1β**
let-7a-5p	15.42	16.11	16.45	15.34	16.18	16.53
let-7b-5p	13.67	15.05	14.02	13.87	14.80	14.39
let-7c	14.30	16.14	14.88	14.92	16.07	15.69
let-7d-5p	18.95	21.11	19.68	18.58	20.04	18.90
let-7e-5p	15.16	16.56	15.88	15.52	17.10	15.73
let-7f-5p	17.30	19.01	19.61	18.19	19.19	19.79
let-7g-5p	17.42	17.55	17.53	17.20	17.82	17.45
miR-100-5p	12.18	12.36	12.73	11.68	11.24	11.56
miR-101-3p	21.53	23.35	23.07	22.13	22.86	23.17
miR-103a-3p	17.08	17.42	17.68	16.74	17.90	16.87
miR-106a-5p	13.41	13.77	13.78	12.71	13.19	12.81
miR-106b-5p	14.70	15.25	16.20	15.09	15.16	15.94
miR-107	21.87	21.92	21.91	21.73	22.40	21.40
miR-10a-5p	16.26	19.72	17.13	16.14	19.02	16.41
miR-10b-5p	19.84	26.56	21.01	19.65	25.76	19.73
miR-125a-3p	25.14	24.51	23.70	23.55	23.56	22.89
miR-125a-5p	17.14	18.08	16.93	16.69	17.10	15.32
miR-125b-5p	8.78	9.07	9.29	9.23	9.46	9.88
miR-126-3p	22.86	23.12	23.59	22.45	23.54	21.79
miR-127-3p	13.67	13.33	13.34	13.39	12.78	13.37
miR-127-5p				24.09	23.34	23.60
miR-128	19.64	20.19	20.38	19.84	20.77	20.90
miR-129-5p	22.68	23.60	21.29	22.58	22.77	22.11
miR-130a-3p	13.58	14.09	14.78	14.04	14.21	14.83
miR-130b-3p	16.14	16.57	16.76	16.61	16.64	16.84
miR-132-3p	13.66	13.62	13.48	13.29	13.49	13.01
miR-133a				21.45	21.53	19.39
miR-135b-5p	24.43	25.82	20.40			
miR-138-5p	13.84	13.57	14.21	14.66	13.47	13.94
miR-139-5p	21.62	21.20	21.37	21.69	21.40	21.47

miR-140-3p	21.94	21.53	20.73	21.35	22.60	20.36
miR-142-3p	24.71	23.81	25.40			
miR-143-3p	15.90	18.20	17.23	15.86	17.82	16.38
miR-145-5p	11.66	12.81	11.84	11.19	12.20	11.48
miR-146a-5p	19.18	16.29	16.90	13.73	11.90	11.77
miR-146b-5p	15.90	15.23	16.37	15.84	15.15	15.53
miR-146b-3p	21.42	20.60	20.89	22.03	21.29	22.32
miR-148a-3p	16.05	18.59	16.68	15.99	18.36	17.09
miR-148b-3p	19.99	20.71	20.87	20.30	20.52	20.98
miR-149-5p	15.66	15.53	15.22	16.07	15.45	15.32
miR-150-5p	22.20	21.29	20.62	22.37	21.27	21.08
miR-152	13.74	14.33	14.35	13.83	14.02	13.99
miR-154-5p	20.55	19.98	21.36	21.08	19.93	21.69
miR-155-5p	18.70	19.37	17.90	16.57	17.71	15.24
miR-15a-5p	19.43	21.63	20.14	19.67	21.22	19.45
miR-15b-5p	15.20	17.26	15.88	15.65	17.14	15.85
miR-16-5p	13.70	14.53	13.89	14.00	14.67	13.55
miR-17-5p	13.36	13.62	13.82	12.67	13.15	12.82
miR-181a-5p	16.15	16.88	16.94	16.03	16.82	16.51
miR-181c-5p	22.11	21.81	21.70	21.92	22.45	22.00
miR-184	21.70	22.51	21.93	22.69	22.53	22.52
miR-185-5p	20.70	20.90	20.84	20.27	20.20	19.96
miR-186-5p	17.69	17.58	17.65	17.10	16.99	16.14
miR-18a-5p	19.99	20.30	21.68	20.34	20.66	21.38
miR-18b-5p	20.02	20.73	21.73	20.43	21.57	20.88
miR-190a	20.67	22.60	21.85	20.98	22.14	21.87
miR-191-5p	11.95	11.84	11.63	11.68	10.91	10.92
miR-192-5p	19.87	19.82	19.17	19.35	19.82	19.68
miR-193a-3p	26.10	24.68	23.40	24.49	25.00	24.73
miR-193a-5p	14.32	13.62	13.94	14.51	13.67	14.45
miR-193b-3p	10.52	10.28	10.50	10.56	10.64	10.72
miR-194-5p	21.84	17.75	20.86			
miR-195-5p	16.61	18.64	17.53	16.68	18.78	16.83

miR-196b-5p	22.69	25.75	21.79	21.30	24.56	20.62
miR-197-3p	13.30	13.29	13.27	14.28	13.81	14.35
miR-198	23.28	23.63	22.05	22.23	21.71	21.64
miR-199a-5p	18.14	18.97	19.08	18.30	18.98	19.38
miR-199a-3p; miR-199b-3p	13.05	14.02	13.91	13.18	14.24	13.27
miR-199b-5p	17.15	18.77	18.55	17.66	18.84	18.68
miR-19a-3p	17.79	17.97	18.54	17.49	18.13	17.80
miR-19b-3p	11.90	11.78	12.46	11.50	11.15	11.64
miR-200a-3p	13.76	14.70	16.78	15.47	15.44	17.37
miR-200b-3p				24.34	24.57	23.89
miR-202-3p	25.96	23.61	18.42	25.86	25.99	25.40
miR-203	20.64	21.27	21.54	22.32	21.76	22.53
miR-204-5p	18.24	16.28	17.36	18.63	16.94	17.94
miR-20a-5p	12.23	12.53	13.11	12.18	12.62	12.88
miR-20b-5p	19.82	20.28	20.74	19.32	21.34	20.29
miR-21-5p	10.60	11.68	11.91	10.98	11.89	11.71
miR-210	15.06	15.08	15.30	13.44	14.55	13.86
miR-211-5p	11.97	14.45	18.48	17.82	18.65	20.48
miR-212-3p	17.91	17.52	17.04	17.42	17.03	16.65
miR-214-3p	12.60	12.82	12.48	13.00	12.69	12.93
miR-215	20.70	20.16	20.94	20.17	19.22	20.30
miR-218-5p	13.23	14.25	13.86	13.66	14.24	13.81
miR-219-5p	24.57	24.49	24.37	24.72	24.42	25.35
miR-219-1-3p	26.75	25.90	26.09	27.39	18.64	26.32
miR-22-3p	15.28	15.96	15.46	15.22	15.07	14.92
miR-221-3p	9.83	9.58	9.97	10.09	9.08	10.08
miR-222-3p	10.13	10.40	9.79	9.60	9.60	8.81
miR-223-3p	22.37	19.91	20.97	22.07	22.17	22.54
miR-224-5p	14.55	15.00	14.91	14.80	14.72	14.84
miR-23a-3p	15.97	16.17	16.38	16.18	15.60	16.69
miR-23b-3p	17.20	18.68	12.89			
miR-24-3p	8.52	8.04	7.95	8.51	7.32	7.76

miR-25-3p	15.66	15.41	15.96	15.61	15.12	15.91
miR-26a-5p	13.41	14.54	14.05	13.65	14.42	13.86
miR-26b-5p	15.62	16.61	16.75	16.08	16.37	16.89
miR-27a-3p	13.75	14.14	15.23	14.62	15.19	15.01
miR-27b-3p	14.54	14.19	15.87	15.65	14.96	15.95
miR-28-3p	15.60	16.64	16.31	15.15	16.22	15.91
miR-28-5p	15.57	16.22	15.25	15.32	15.39	14.77
miR-296-5p	15.38	15.08	15.56	15.92	14.98	16.41
miR-296-3p	22.46	22.07	20.60	21.53	21.57	21.36
miR-299-5p	22.14	22.45	23.12	22.66	22.10	22.63
miR-29a-3p	13.31	14.14	13.95	13.54	13.80	12.97
miR-29b-3p	16.98	18.12	18.78	16.94	17.48	18.10
miR-29c-3p	14.54	15.24	15.84	14.68	15.72	15.27
miR-301a-3p	17.75	18.02	18.38	17.86	18.46	18.34
miR-301b	22.11	22.83	23.19	22.64	22.44	23.33
miR-302a-3p	10.63	11.23	22.47	27.43	18.24	25.82
miR-302c-3p	25.81	21.95	21.30	26.08	14.17	27.90
miR-30b-5p	11.22	11.03	11.87	11.96	11.13	12.35
miR-30c-5p	11.15	10.92	11.44	11.71	11.08	11.87
miR-31-5p	11.90	12.33	12.06	11.46	11.37	10.83
miR-320a	13.18	12.43	12.87	13.30	12.39	12.89
miR-323-3p	20.21	19.36	20.21	19.98	18.67	19.77
miR-324-3p	18.71	19.17	18.93	18.37	18.21	17.48
miR-324-5p	16.36	16.99	17.01	16.51	17.00	17.15
miR-326				20.14	21.79	21.50
miR-328	12.70	12.53	12.63	13.61	13.18	13.92
miR-329	21.72	21.38	23.41	22.45	21.18	22.77
miR-330-3p	19.71	20.79	19.38	20.23	20.31	19.61
miR-331-3p	14.17	15.54	14.27	14.38	15.20	14.08
miR-331-5p	22.88	15.68	21.49	9.86	21.13	19.80
miR-335-5p	16.99	15.16	17.40	18.05	15.91	17.90
miR-337-5p	21.37	20.26	21.63	20.89	20.13	20.86
miR-339-3p	20.87	20.57	20.28	20.15	19.97	19.66

miR-339-5p	15.98	15.80	15.83	15.61	15.23	15.58
miR-340-5p	24.33	26.27	26.01	24.53	26.35	25.15
miR-342-3p	16.31	15.58	15.38	15.70	15.15	14.46
miR-345-5p	18.61	18.41	18.39	17.92	17.97	17.41
miR-346	17.21	17.75	17.86	17.09	17.20	18.51
miR-34a-5p	12.57	12.84	13.08	12.79	13.16	13.73
miR-34c-5p	16.81	17.33	17.66	17.55	17.24	18.25
miR-361-5p	16.49	16.62	16.72	16.56	16.29	16.94
miR-362-5p	23.74	23.34	22.01	22.40	23.54	21.89
miR-362-3p	23.49	24.59	26.24	23.57	23.93	25.25
miR-365a-3p; miR-365b-3p	14.72	15.71	15.81	15.22	20.27	21.43
miR-367-3p	12.67	18.22	14.33	23.56	17.09	19.14
miR-369-3p	22.91	23.54	23.93	23.50	23.49	23.81
miR-370	16.03	15.58	15.35	15.74	15.43	15.40
miR-372	23.69	24.73	23.48	23.26	23.84	23.89
miR-373-3p	21.79	18.51	21.60	22.16	22.16	22.74
miR-374a-5p	16.98	19.22	18.37	17.65	19.00	18.27
miR-375	25.82	24.62	24.38	26.59	24.74	24.92
miR-376a-3p	16.33	15.26	16.60	15.48	14.43	15.38
miR-376b				23.07	21.96	23.26
miR-376c	16.46	15.57	16.50	16.14	15.50	15.79
miR-377-3p				21.93	23.05	23.61
miR-380-3p				23.45	22.68	24.04
miR-381	21.70	21.03	21.48	21.88	21.21	22.15
miR-382-5p	13.44	13.04	13.92	13.61	13.18	13.88
miR-383	20.70	19.48	20.59	20.86	20.41	21.90
miR-409-5p	21.80	21.39	22.53	21.47	21.90	21.97
miR-410	19.31	18.64	19.79	19.20	18.85	19.15
miR-411-5p	17.85	19.12	18.43	17.51	17.98	17.49
miR-422a	24.38	23.26	23.80	24.79	23.44	24.04
miR-423-5p	16.98	17.19	16.90	17.56	17.32	18.24
miR-424-5p	18.62	20.96	19.69	18.86	20.93	18.98

miR-425-5p	17.54	17.98	16.85	18.02	16.14	16.92
miR-431-5p				18.69	17.86	17.67
miR-433	19.89	19.64	19.29	20.63	19.47	20.31
miR-449b-5p				25.63	25.66	25.11
miR-450a-5p	23.95	26.11	23.44	23.61	26.37	24.18
miR-452-5p	20.43	21.83	21.52	20.43	21.17	20.28
miR-454-3p	22.44	23.56	22.05	21.50	20.92	17.03
miR-455-5p	20.25	20.22	20.35	19.64	19.83	20.05
miR-455-3p	19.01	19.08	18.85	19.16	18.53	19.18
miR-483-5p	16.68	17.32	14.79	17.96	16.27	18.46
miR-484	13.13	12.61	12.73	12.65	12.21	11.91
miR-485-3p	20.68	20.29	20.88	21.14	20.70	21.13
miR-485-5p				19.14	16.17	19.67
miR-487a	23.18	22.88	22.57	23.29	22.88	22.75
miR-487b	21.18	20.11	18.20	20.95	20.16	20.35
miR-489				24.67	24.87	24.45
miR-493-3p	23.51	22.66	22.31	21.85	22.80	21.67
miR-494	18.65	17.28	20.17	18.23	16.52	17.22
miR-500a-5p	21.25	21.57	20.49	21.94	23.16	21.71
miR-501-5p	20.34	22.50	21.84	19.91	15.80	19.91
miR-501-3p	22.26	18.62	22.28	21.04	20.02	21.10
miR-502-5p	21.93	23.11	19.52	22.50	23.58	22.19
miR-502-3p	21.63	22.52	21.10	21.74	22.14	22.26
miR-503	19.72	20.16	20.60	18.86	20.02	18.53
miR-505-3p	20.68	20.10	19.93	20.66	20.81	22.07
miR-509-5p	20.27	19.27	24.31	19.94	19.75	21.72
miR-511				23.63	17.86	24.00
miR-512-3p	20.42	19.64	24.78	21.57	20.96	22.14
miR-517a-3p; miR-517b-3p			25.22	25.46	25.23	
miR-517c-3p	24.73	22.97	14.84	25.52	25.23	25.90
miR-518d-3p	23.47	22.95	22.58			
miR-518f-3p	5.12	12.59	24.47			

miR-519a-3p				25.53	24.83	21.45
miR-520b				21.69	22.27	16.90
miR-520e	11.74	23.31	22.86	22.36	11.37	16.35
miR-520f	25.66	21.27	16.08	24.42	24.87	26.17
miR-523-3p				13.49	11.76	12.09
miR-526b-5p	22.56	22.04	17.35	23.02	22.12	23.19
miR-532-5p	16.04	16.41	20.72	16.01	16.00	15.88
miR-532-3p	17.76	18.55	23.94	17.61	17.93	16.89
miR-539-5p	20.86	20.23	23.24	20.09	20.05	19.33
miR-541-3p				23.16	23.63	19.24
miR-542-3p	22.66	23.84	22.77	23.25	24.13	24.33
miR-542-5p	22.96	22.31	26.45	23.05	22.70	21.68
miR-545-3p	24.71	26.44	19.66	25.12	24.82	24.59
miR-548a-3p	25.11	25.63	20.89	25.40	27.39	27.65
miR-548b-3p	20.54	19.86	19.91	20.73	21.25	20.75
miR-548am-5p;	21.32	20.67	8.63	21.43	20.20	22.34
miR-548d-5p	21.09	21.27	27.47	21.65	21.42	20.94
miR-551b-3p	27.32	5.45	12.36	6.31	26.80	14.09
miR-574-3p	12.83	11.93	22.06	12.67	12.02	12.04
miR-576-3p	22.41	21.98	24.40	21.76	22.15	22.20
miR-576-5p	24.20	21.50	19.44	24.49	22.98	23.17
miR-579	22.31	23.58	22.02	22.83	23.89	24.34
miR-590-5p	18.92	18.77	21.08	18.67	18.14	18.54
miR-597	21.63	22.42	21.78	21.72	22.91	22.24
miR-615-5p	22.34	22.44	24.60	21.13	21.66	20.60
miR-616-3p	22.81	22.35	24.16	22.66	22.19	22.01
miR-625-5p	24.72	26.03	25.04	24.73	25.85	23.87
miR-628-5p	24.30	26.20	17.87	24.48	26.97	22.97
miR-629-5p	24.34	25.30	24.19	25.12	25.34	24.49
miR-636	20.34	17.88	22.05	19.30	17.36	16.59
miR-642a-5p	23.87	24.28	15.89	23.64	23.16	23.96
miR-652-3p	21.70	21.92	24.44	21.10	21.87	20.81
miR-654-5p	16.59	15.87	26.49	16.93	16.24	16.24

miR-654-3p	23.66	22.50	17.65	23.69	22.44	24.35
miR-655	23.07	21.87	19.91	22.98	24.09	24.59
miR-660-5p	17.07	17.57	18.66	17.11	17.37	17.62
miR-671-3p	21.13	20.81	17.67	20.67	19.23	19.88
miR-708-5p	19.48	20.48	21.39	18.72	20.22	17.82
miR-744-5p	17.75	18.16	22.85	17.47	17.40	16.73
miR-758	21.44	20.60	13.52	21.92	21.32	21.56
miR-885-5p	23.49	22.72	13.89	23.96	23.65	23.39
miR-886-3p (v15)	15.48	15.64	24.84	13.43	13.23	12.06
miR-886-5p (v15)	16.41	16.64	24.14	14.83	14.54	12.50
miR-888-5p	24.65	19.56	21.07	15.97	19.15	23.93
miR-889	22.14	22.49	22.95	22.49	22.30	23.96
miR-9-5p	20.22	22.10	11.82	23.73	22.72	22.96
miR-92a-3p	22.67	21.91	22.38	22.55	22.38	22.74
miR-95	11.67	11.05	20.25	11.77	11.15	12.59
miR-98	21.66	25.32	12.87	18.71	18.50	19.89
miR-99a-5p	17.95	20.49	13.25	11.54	11.20	11.76
miR-99b-5p	12.20	12.15	21.64	12.73	13.69	13.32
miR-124-3p	12.70	14.03	19.73	21.82	19.49	22.64
miR-129-2-3p	20.13	20.21	17.37	19.07	19.00	19.92
miR-134	19.70	19.51	19.27	17.64	16.82	16.54
miR-137	18.06	17.09	16.65	18.60	19.18	19.43
miR-140-5p	17.65	18.60	19.22	16.00	17.39	15.83
miR-187-3p	16.18	17.58	19.39			
miR-374b-5p	17.48	19.44	23.32	18.53	20.08	18.27
miR-379-5p	18.66	19.57	18.87	18.68	19.15	18.46
miR-451a	24.19	22.39	19.43	24.72	24.15	24.88
miR-491-5p	19.81	19.60	28.01	19.61	19.30	18.56
miR-495	18.22	17.63	14.35	18.39	18.17	18.85
miR-496				25.72	24.20	13.53
miR-499a-5p	26.67	24.37	16.64			
miR-615-3p	15.96	14.49	14.35	16.52	17.20	15.68
miR-93-5p	16.49	16.23	16.64	16.17	15.97	27.66

miR-7-5p	23.91	25.44	24.17	22.77	23.80	24.20
let-7f-1-3p	16.50	24.88	25.11			
let-7f-2-3p				27.91	25.60	26.72
let-7i-3p				26.10	26.02	26.80
miR-100-3p				25.84	28.54	26.20
miR-106b-3p	19.50	18.64	19.46	18.61	18.01	18.27
miR-10a-3p				25.54	27.28	27.09
miR-10b-3p	20.37	23.03	20.96	20.54	23.08	20.12
hsmiR-1180	21.46	21.52	21.37	21.61	22.89	22.74
miR-1183	14.64	17.04	17.74	18.50	20.00	18.66
miR-1197	25.90	25.92	26.01	26.61	25.71	28.56
miR-1208	16.47	9.17	17.94	12.64	12.41	14.81
miR-122-3p	15.37	15.86	15.26			
miR-1226-5p	21.64	22.59	21.54	22.75	21.83	21.26
miR-1227	21.20	21.32	19.90	22.37	20.70	20.44
miR-1233	16.30	17.11	15.87	18.18	17.02	17.14
miR-1244	25.37	25.39	24.96	25.94	24.63	25.10
miR-1247-5p	20.80	21.27	19.17	20.67	20.06	31.95
miR-1254	23.78	23.72	21.08	22.88	22.83	22.46
miR-1255b-5p	20.80	20.68	20.41	21.90	20.50	21.81
miR-125b-1-3p	21.28	21.38	21.19	20.62	21.43	20.79
miR-125b-2-3p	21.51	21.16	22.24	22.74	21.87	21.75
miR-126-5p	24.92	24.07	24.68	24.97	24.35	24.73
miR-1260a	15.71	16.59	15.27	15.96	15.93	15.53
miR-1265	25.38	23.34	23.61	26.04	24.46	25.03
miR-1267	14.43	20.62	11.84	16.82	14.02	13.26
miR-1270	22.76	14.90	21.90	24.30	24.11	15.71
miR-1271-5p	21.53	21.20	21.02	20.63	20.53	19.81
miR-1275	16.25	16.43	15.59	16.60	16.50	15.31
miR-1276	24.09	23.71	23.01	23.95	25.17	24.03
miR-1282				23.82	25.46	24.10
miR-1285-3p	21.04	21.00	20.09	20.99	21.65	19.84
miR-1290				20.46	20.30	19.08

miR-1291	18.49	18.88	18.97	17.82	18.71	18.42
miR-1296	18.41	17.97	16.85	18.47	18.42	18.51
miR-1300 (v13)	23.67	23.84	22.96	23.87	24.10	23.69
miR-1303	19.75	19.75	18.14	19.29	18.65	18.89
miR-1304-5p				18.96	18.75	17.53
miR-130b-5p	24.22	19.05	21.67			
miR-1324				26.97	26.04	26.33
miR-132-5p	23.19	22.75	22.91			
miR-135b-3p	18.75	18.67	21.18	18.70	16.93	18.79
miR-136-3p	22.44	21.82	22.08	22.07	21.49	21.63
miR-145-3p	23.67	25.27	24.18	23.83	24.37	23.76
miR-151a-3p	19.26	19.15	19.27	18.66	18.60	18.20
miR-151a-5p	22.04	22.42	21.52	21.69	22.76	21.22
miR-154-3p	23.95	25.08	25.73	24.69	25.27	26.13
miR-15a-3p	24.53	24.39	21.35	24.38	24.34	25.30
miR-15b-3p				20.85	21.84	21.60
miR-16-1-3p	24.53	24.68	24.50	24.78	26.01	24.76
miR-181a-3p	23.35	22.30	22.95	22.83	21.89	22.03
miR-181c-3p				22.87	22.65	22.47
miR-183-3p	24.18	23.09	22.96	23.74	22.93	23.02
miR-18a-3p	26.91	26.03	25.66	26.47	25.50	25.09
miR-190b	27.76	26.17	26.66	25.52	26.04	26.34
miR-191-3p	25.33	24.90	23.12	26.19	26.08	24.61
miR-193b-5p	19.86	19.85	19.23	19.93	20.60	20.74
miR-19b-1-5p	23.82	24.27	23.57	23.64	23.79	23.90
miR-206	24.39	23.69	25.44	25.38	25.47	25.75
miR-20a-3p	25.96	26.43	27.49	26.19	28.44	25.81
miR-21-3p	22.08	22.33	22.36	21.89	22.35	22.72
miR-181a-3p	23.89	22.77	23.26	23.09	21.98	22.74
miR-214-5p	20.50	20.61	20.69	20.66	21.97	20.03
miR-218-2-3p	26.75	26.99	26.04	26.35	26.12	25.41
miR-22-5p	19.26	20.66	20.34	19.94	21.11	20.08
miR-221-5p	24.09	25.41	25.38	24.88	25.82	24.83

miR-222-5p	20.49	19.37	19.56	18.47	18.00	18.26
miR-223-5p	25.26	24.58	25.73			
miR-23a-5p	23.52	24.40	24.32	24.05	23.76	24.46
miR-24-2-5p	20.65	20.69	21.36	21.31	20.83	21.21
miR-27a-5p	22.50	22.64	22.14	22.55	22.28	21.44
miR-27b-5p	23.62	23.49	23.77	23.64	24.11	23.51
miR-29a-5p	18.17	18.33	19.20	18.32	18.45	19.75
miR-29b-2-5p				27.23	25.76	26.82
miR-302d-3p	26.08	26.42	25.04			
miR-30a-3p	16.45	16.27	17.77	16.34	15.72	17.09
miR-30a-5p	14.43	14.21	15.07	15.28	14.91	15.23
miR-30d-3p	24.62	24.38	24.38	24.73	24.13	25.55
miR-30d-5p	17.22	16.87	17.49	18.03	17.34	17.80
miR-30e-3p	16.32	15.99	17.66	16.46	15.72	17.09
miR-31-3p	15.50	15.12	16.49	15.52	15.36	15.93
miR-320b	19.06	18.39	19.42	19.26	18.31	19.46
miR-335-3p	22.04	20.37	21.71	22.65	22.18	22.18
miR-337-3p	24.18	23.33	23.94	24.63	22.96	24.18
miR-338-5p	24.50	24.86	26.17	25.62	26.12	23.33
miR-33a-3p	24.98	25.76	23.59	26.21	24.48	26.31
miR-340-3p	27.05	26.48	26.62	26.54	27.90	26.38
miR-34a-3p	17.32	17.93	17.81	17.72	17.85	18.05
miR-34b-5p	21.53	22.11	22.72	22.62	22.44	23.51
miR-34b-3p	18.80	18.14	18.31	18.97	18.07	18.06
miR-361-3p				22.09	21.23	21.06
miR-377-5p	24.52	23.21	24.69	24.53	23.86	23.65
miR-378a-5p	24.96	23.52	24.31	25.07	25.14	24.75
miR-378a-3p	23.76	22.57	22.71			
miR-380-5p	23.74	24.69	23.56	23.71	22.72	22.76
miR-409-3p	15.01	14.36	14.31	14.70	13.67	13.73
miR-411-3p	25.53	24.42	25.58	25.23	28.46	25.17
miR-424-3p	19.48	20.38	19.49	19.85	20.50	18.78
miR-425-3p	23.76	23.16	22.45	24.12	23.89	23.08

miR-432-3p	22.27	21.85	22.59	23.38	24.94	22.34
miR-432-5p	21.93	20.38	20.82	21.56	20.14	20.35
miR-483-3p				22.54	16.42	18.47
miR-488-5p	23.51	22.85	23.27	23.57	23.51	24.18
miR-497-5p	21.38	22.18	21.40	22.31	22.71	22.56
miR-500a-3p	23.07	26.24	20.46	24.19	18.79	26.19
miR-505-5p	23.39	22.74	22.41	24.20	23.12	22.51
miR-516a-3p; miR-516b-3p	24.47	24.33	23.91	25.47	26.05	24.15
miR-519b-3p	29.49	18.53	15.84	28.01	27.74	19.48
miR-520c-3p	26.06	24.07	26.32	19.81	20.49	23.17
miR-520d-3p	28.41	25.43	26.67	19.23	26.26	25.76
miR-541-5p	24.34	19.21	22.83	23.00	21.84	22.96
miR-543	21.12	20.75	21.08	21.14	20.33	21.22
miR-550a-5p				26.14	26.55	26.78
miR-551b-5p				24.18	21.50	19.50
miR-564	19.96	18.66	19.81	19.71	18.72	19.82
miR-566	17.11	15.64	16.30			
miR-572	21.35	21.49	20.65	21.33	21.36	21.32
miR-589-3p	22.48	22.28	23.28			
miR-590-3p	25.32	25.99	26.55	25.45	25.45	24.72
miR-591	25.25	22.61	24.88	25.67	25.38	24.31
miR-601	23.46	24.41	22.67	23.29	22.66	23.70
miR-604				26.65	25.67	26.11
miR-605	20.88	21.09	20.24	21.75	21.51	20.81
miR-616-5p	24.36	23.53	22.88	24.27	24.14	23.59
miR-622	23.98	24.51	23.93	24.74	24.68	24.85
miR-623	19.22	20.04	19.67			
miR-624-5p	24.53	19.56	24.62	24.91	25.72	22.21
miR-625-3p	21.17	21.15	20.32	21.21	21.80	19.97
miR-628-3p	25.65	25.41	24.18			
miR-629-3p	22.82	23.23	21.71	22.66	22.85	22.07
miR-635	24.51	24.40	24.75	23.95	24.11	24.01

miR-638	19.89	19.32	19.42	20.05	19.71	19.99
miR-639				21.13	17.14	21.82
miR-645	23.22	22.84	22.74	23.40	22.09	23.61
miR-646				10.98	11.09	11.07
miR-648	18.13	19.41	20.38	19.02	16.36	21.29
miR-650	20.45	21.26	17.66	20.52	20.21	19.89
miR-656	21.47	21.88	22.35	23.60	23.62	23.80
miR-659-3p	19.49	18.89	19.13	19.94	18.26	20.15
miR-661	17.45	17.03	16.45	17.30	16.99	16.51
miR-662	18.93	18.69	17.42	18.98	16.52	17.52
miR-663b	15.63	15.15	16.07	15.64	14.93	15.69
miR-664-3p	19.17	19.44	18.50	19.93	20.45	17.93
miR-7-2-3p				24.64	24.20	23.78
miR-720	10.75	12.84	12.44	11.01	11.06	10.66
miR-744-3p	23.53	23.33	22.78	23.44	22.92	22.64
miR-765	22.86	20.54	22.72	24.24	19.68	22.57
miR-766-3p	18.44	17.63	17.41	17.59	18.57	17.91
miR-769-5p	21.11	20.75	20.94	21.15	21.24	21.05
miR-770-5p	12.65	15.25	14.57	14.75	12.95	15.41
miR-92a-1-5p				27.13	28.33	19.99
miR-93-3p	22.13	21.34	21.84	21.58	21.09	20.71
miR-939	17.03	15.97	14.69	15.61	17.01	15.25
miR-942	20.93	21.41	20.68	20.95	21.03	20.59
miR-943	22.12	22.79	21.45	22.54	22.09	21.69
miR-99a-3p	22.51	23.93	22.63	22.92	23.83	23.28
miR-99b-3p	21.98	21.96	21.05	22.62	21.86	21.46
miR-29c-5p	21.50	22.52	22.27	24.64	15.92	22.92
miR-7-1-3p	18.46	18.21	18.38	18.44	18.02	17.82
