# Supplementary material for: Adipose-Derived Mesenchymal Stromal Cells Treated with Interleukin 1 Beta Produced Chondro-Protective Vesicles Able to Fast Penetrate in Cartilage
Source: Cells. 2021 May 12;10(5):1180. doi: 10.3390/cells10051180 (PMC8151616; doi:10.3390/cells10051180)
Supplement: Supplementary file 1 [file cells-10-01180-s001.zip › Table S3.pdf]

Table S3: target genes of miRNAs activated in ASCs treated with IL-1β.

| Gene Symbol | p-value | Interactions | miRNAs |
|---|---|---|---|
| GIGYF1 | 0.045994 | 7 | miR-449b-5p, miR-485-5p, miR-377-3p, miR-541-3p, miR-604, miR-511-5p, miR-1324 |
| SHOC2 | 0.007467 | 7 | miR-519a-3p, miR-380-3p, miR-200b-3p, let-7f-2-3p, miR-127-5p, miR-449b-5p, miR-485-5p |
| SETD1B | 0.038156 | 5 | miR-127-5p, miR-92a-1-5p, miR-646, miR-489-3p, miR-541-3p |
| E2F3 | 0.031531 | 5 | miR-200b-3p, miR-449b-5p, miR-100-3p, miR-376b-3p, miR-377-3p |
| SESN3 | 0.024922 | 5 | miR-511-5p, miR-431-5p, let-7f-2-3p, miR-133a-3p, miR-519a-3p |
| MAZ | 0.021988 | 5 | miR-326, miR-92a-1-5p, miR-604, miR-449b-5p, miR-483-3p |
| RORA | 0.018655 | 5 | let-7f-2-3p, miR-127-5p, miR-1290, miR-483-3p, miR-377-3p |
| RPP14 | 0.003831 | 5 | miR-326, miR-511-5p, miR-376b-3p, miR-431-5p, miR-519a-3p |
| ADM | 0.003241 | 5 | miR-127-5p, miR-485-5p, miR-541-3p, miR-29b-2-5p, miR-380-3p |
| ACTB | 0.053328 | 4 | miR-326, miR-127-5p, let-7i-3p, miR-7-2-3p |
| FEM1B | 0.053328 | 4 | miR-646, miR-519a-3p, miR-489-3p, miR-511-5p |
| FGF2 | 0.050340 | 4 | miR-181c-3p, miR-7-2-3p, miR-646, miR-376b-3p |
| TMBIM6 | 0.048883 | 4 | miR-551b-5p, miR-485-5p, miR-1304-5p, miR-511-5p |
| IGF1 | 0.040646 | 4 | miR-483-3p, miR-10a-3p, miR-485-5p, miR-133a-3p |
| SMAD4 | 0.029920 | 4 | miR-483-3p, miR-519a-3p, miR-551b-5p, miR-449b-5p |
| ZNF704 | 0.026774 | 4 | miR-511-5p, miR-431-5p, miR-646, miR-133a-3p |
| DLC1 | 0.021110 | 4 | miR-200b-3p, miR-646, miR-29b-2-5p, miR-483-3p |
| IRF2BP2 | 0.021110 | 4 | miR-551b-5p, miR-29b-2-5p, let-7f-2-3p, miR-431-5p |
| SERPINH1 | 0.018585 | 4 | miR-361-3p, miR-1304-5p, miR-200b-3p, miR-133a-3p |
| TJAP1 | 0.017013 | 4 | miR-92a-1-5p, miR-483-3p, miR-200b-3p, miR-377-3p |
| STX16 | 0.016260 | 4 | miR-100-3p, miR-29b-2-5p, miR-550a-5p, miR-200b-3p |
| BLCAP | 0.012185 | 4 | miR-431-5p, miR-519a-3p, miR-10a-3p, miR-100-3p |
| PTMA | 0.088445 | 4 | miR-7-2-3p, miR-377-3p, miR-133a-3p, miR-92a-1-5p |
| PLEKHA3 | 0.039358 | 4 | miR-133a-3p, miR-541-3p, miR-15b-3p, miR-377-3p |
| KSR2 | 0.003260 | 4 | miR-361-3p, miR-483-3p, miR-511-5p, miR-1324 |
| FRS2 | 0.023838 | 4 | miR-10a-3p, miR-519a-3p, miR-483-3p, miR-200b-3p |
| TRIM38 | 0.001073 | 4 | miR-489-3p, miR-29b-2-5p, miR-1304-5p, miR-431-5p |
| BBX | 0.053270 | 3 | miR-519a-3p, miR-29b-2-5p, miR-200b-3p |

| | | | |
|---|---|---|---|
| PCDHA6 | 0.053270 | 3 | miR-511-5p, miR-431-5p, miR-485-5p |
| FAM210A | 0.051236 | 3 | miR-519a-3p, miR-29b-2-5p, miR-92a-1-5p |
| PTPN4 | 0.051236 | 3 | miR-519a-3p, miR-489-3p, miR-200b-3p |
| ZBTB4 | 0.051236 | 3 | miR-483-3p, miR-431-5p, miR-519a-3p |
| KLF6 | 0.049242 | 3 | miR-550a-5p, miR-7-2-3p, miR-511-5p |
| ZNF439 | 0.049242 | 3 | miR-376b-3p, miR-1304-5p, miR-485-5p |
| MXRA7 | 0.047286 | 3 | miR-15b-3p, let-7i-3p, miR-485-5p |
| NRXN3 | 0.047286 | 3 | miR-1304-5p, miR-485-5p, miR-1282 |
| FZR1 | 0.045371 | 3 | miR-29b-2-5p, miR-449b-5p, miR-541-3p |
| ZNF644 | 0.045371 | 3 | miR-604, miR-449b-5p, miR-7-2-3p |
| AASDHPPT | 0.043497 | 3 | miR-377-3p, let-7f-2-3p, miR-449b-5p |
| RAB3B | 0.041662 | 3 | miR-200b-3p, miR-127-5p, miR-483-3p |
| SEC61A1 | 0.041662 | 3 | miR-1324, miR-449b-5p, miR-646 |
| PPP1R15B | 0.404100 | 3 | miR-519a-3p, miR-550a-5p, miR-485-5p |
| PTBP3 | 0.039868 | 3 | miR-15b-3p, miR-377-3p, miR-29b-2-5p |
| LIN7C | 0.038115 | 3 | miR-127-5p, miR-646, miR-551b-5p |
| PSD3 | 0.038115 | 3 | miR-326, miR-200b-3p, miR-519a-3p |
| KIF13A | 0.036404 | 3 | let-7f-2-3p, miR-519a-3p, miR-200b-3p |
| NLGN4X | 0.034733 | 3 | miR-1324, miR-200b-3p, miR-7-2-3p |
| PLEKHA6 | 0.029970 | 3 | miR-377-3p, miR-485-5p, miR-1324 |
| SRRM4 | 0.021572 | 3 | miR-511-5p, miR-431-5p, miR-604 |
| CCNE2 | 0.019108 | 3 | miR-449b-5p, miR-200b-3p, miR-646 |
| SLITRK4 | 0.017938 | 3 | miR-10a-3p, miR-1324, miR-377-3p |
| SLIT1 | 0.015724 | 3 | miR-541-3p, miR-485-5p, miR-1324 |
| PSMG1 | 0.014678 | 3 | miR-133a-3p, miR-483-3p, miR-646 |
| SOX12 | 0.014678 | 3 | miR-541-3p, miR-29b-2-5p, miR-1324 |
| CKS1B | 0.011788 | 3 | miR-1304-5p, miR-100-3p, miR-485-5p |
| RS1 | 0.011788 | 3 | miR-376b-3p, let-7f-2-3p, miR-646 |
| C1orf52 | 0.010062 | 3 | miR-1304-5p, let-7f-2-3p, miR-431-5p |
| HOXD9 | 0.009259 | 3 | miR-551b-5p, miR-361-3p, miR-29b-2-5p |

| | | | |
|---|---|---|---|
| EML4 | 0.007770 | 3 | miR-551b-5p, let-7f-2-3p, miR-377-3p |
| NCBP2 | 0.005821 | 3 | miR-10a-3p, miR-7-2-3p, miR-431-5p |
| MOCS2 | 0.004201 | 3 | miR-7-2-3p, miR-551b-5p, miR-519a-3p |
| CACNA1B | 0.003731 | 3 | miR-377-3p, miR-1304-5p, miR-15b-3p |
| EPB42 | 0.003731 | 3 | miR-361-3p, miR-380-3p, miR-431-5p |
| KLHL20 | 0.003295 | 3 | miR-200b-3p, miR-511-5p, miR-431-5p |
| ZNF202 | 0.003295 | 3 | miR-15b-3p, miR-1324, miR-519a-3p |
| FAM46A | 0.028466 | 3 | miR-511-5p, miR-431-5p, miR-489-3p |
| PPM1A | 0.028466 | 3 | miR-377-3p, miR-376b-3p, miR-646 |
| USP48 | 0.028466 | 3 | let-7i-3p, miR-646, miR-1290 |
| PABPC1 | 0.027003 | 3 | miR-485-5p, miR-200b-3p, miR-7-2-3p |
| POU2F2 | 0.027003 | 3 | miR-29b-2-5p, miR-133a-3p, miR-92a-1-5p |
| ZNF525 | 0.022867 | 3 | miR-127-5p, miR-100-3p, miR-511-5p |
| MED19 | 0.000911 | 3 | miR-1324, miR-100-3p, miR-551b-5p |
| GOT1 | 0.005246 | 3 | miR-1324, miR-483-3p, miR-200b-3p |
| ALX1 | 0.001871 | 3 | miR-485-5p, miR-127-5p, miR-431-5p |
| ANO8 | 0.053518 | 2 | miR-1282, miR-1290 |
| CHST12 | 0.053518 | 2 | miR-541-3p, miR-483-3p |
| FAM216B | 0.053518 | 2 | miR-326, miR-200b-3p |
| GOSR2 | 0.053518 | 2 | miR-377-3p, miR-326 |
| RASSF8 | 0.053518 | 2 | miR-200b-3p, miR-511-5p |
| SMURF2 | 0.053518 | 2 | miR-511-5p, miR-431-5p |
| C1orf147 | 0.050111 | 2 | let-7f-2-3p, miR-483-3p |
| NFATC2 | 0.050111 | 2 | miR-10a-3p, miR-489-3p |
| ZFHX4 | 0.050111 | 2 | miR-200b-3p, miR-646 |
| ZNF781 | 0.050111 | 2 | miR-376b-3p, miR-377-3p |
| CDK17 | 0.046787 | 2 | miR-1324, miR-646 |
| FUT4 | 0.046787 | 2 | miR-489-3p, miR-10a-3p |
| MAP2K6 | 0.046787 | 2 | miR-1304-5p, miR-1324 |
| PHLPP1 | 0.046787 | 2 | miR-200b-3p, miR-7-2-3p |

| | | | |
|---|---|---|---|
| PSMC4 | 0.046787 | 2 | miR-133a-3p, miR-485-5p |
| ZHX3 | 0.046787 | 2 | miR-100-3p, miR-483-3p |
| FZD6 | 0.463568 | 2 | miR-519a-3p, miR-550a-5p |
| LTBP4 | 0.043550 | 2 | miR-1304-5p, miR-523-3p |
| PCMT1 | 0.043550 | 2 | miR-646, miR-7-2-3p |
| ZNF131 | 0.043550 | 2 | miR-29b-2-5p, miR-1290 |
| C8orf4 | 0.040402 | 2 | miR-1290, miR-380-3p |
| DNAJC3 | 0.040402 | 2 | miR-485-5p, miR-127-5p |
| DUSP6 | 0.040402 | 2 | miR-377-3p, let-7f-2-3p |
| LOX | 0.040402 | 2 | miR-200b-3p, miR-511-5p |
| ZNF736 | 0.040402 | 2 | miR-376b-3p, miR-7-2-3p |
| ADAM12 | 0.037347 | 2 | miR-200b-3p, miR-1304-5p |
| C8orf17 | 0.037347 | 2 | miR-326, miR-181c-3p |
| EGF | 0.037347 | 2 | miR-1304-5p, miR-485-5p |
| FERMT2 | 0.037347 | 2 | miR-200b-3p, miR-1324 |
| ITCH | 0.037347 | 2 | miR-127-5p, miR-485-5p |
| KLF9 | 0.037347 | 2 | miR-511-5p, miR-431-5p |
| MAOB | 0.037347 | 2 | miR-1290, miR-361-3p |
| NAPEPLD | 0.037347 | 2 | miR-127-5p, miR-100-3p |
| NDFIP2 | 0.037347 | 2 | miR-489-3p, miR-551b-5p |
| C5orf22 | 0.031522 | 2 | miR-551b-5p, miR-15b-3p |
| FOXG1 | 0.031522 | 2 | miR-376b-3p, miR-200b-3p |
| UBE4A | 0.031522 | 2 | miR-29b-2-5p, miR-326 |
| ZMYM4 | 0.031522 | 2 | miR-449b-5p, miR-551b-5p |
| ZNF274 | 0.031522 | 2 | miR-1324, miR-483-3p |
| FOXH1 | 0.026102 | 2 | miR-550a-5p, miR-361-3p |
| SYT9 | 0.026102 | 2 | miR-127-5p, miR-100-3p |
| ZNF25 | 0.026102 | 2 | miR-489-3p, miR-431-5p |
| A4GALT | 0.023552 | 2 | miR-483-3p, miR-326 |
| CEP126 | 0.023552 | 2 | miR-1304-5p, miR-485-5p |

| | | | |
|---|---|---|---|
| LPIN1 | 0.023552 | 2 | miR-485-5p, miR-489-3p |
| LRFN1 | 0.023552 | 2 | miR-485-5p, miR-361-3p |
| TBK1 | 0.023552 | 2 | let-7f-2-3p, miR-200b-3p |
| TMC5 | 0.023552 | 2 | miR-511-5p, miR-431-5p |
| ZNF764 | 0.023552 | 2 | miR-377-3p, miR-541-3p |
| KCTD16 | 0.021111 | 2 | miR-377-3p, miR-483-3p |
| PAQR8 | 0.021111 | 2 | miR-326, miR-361-3p |
| ZFP64 | 0.021111 | 2 | miR-361-3p, miR-483-3p |
| FOXF2 | 0.018785 | 2 | miR-519a-3p, miR-377-3p |
| MYEF2 | 0.018785 | 2 | miR-551b-5p, miR-7-2-3p |
| RRAS | 0.018785 | 2 | miR-1304-5p, miR-485-5p |
| CNTF | 0.014487 | 2 | miR-377-3p, miR-551b-5p |
| FXYD6 | 0.014487 | 2 | miR-377-3p, miR-541-3p |
| GBF1 | 0.014487 | 2 | miR-485-5p, miR-377-3p |
| GPR89A | 0.014487 | 2 | miR-511-5p, miR-431-5p |
| GPR89B | 0.014487 | 2 | miR-511-5p, miR-431-5p |
| WARS2 | 0.014487 | 2 | miR-646, miR-551b-5p |
| PAX1 | 0.012523 | 2 | miR-29b-2-5p, miR-646 |
| VKORC1 | 0.012523 | 2 | miR-133a-3p, miR-326 |
| CRB2 | 0.010686 | 2 | miR-377-3p, miR-483-3p |
| LYPD3 | 0.010686 | 2 | miR-1304-5p, miR-377-3p |
| NGRN | 0.010686 | 2 | miR-200b-3p, miR-100-3p |
| EPHB3 | 0.007410 | 2 | miR-377-3p, miR-326 |
| PRR15 | 0.007410 | 2 | miR-361-3p, miR-483-3p |
| C8A | 0.057005 | 2 | miR-127-5p, miR-483-3p |
| DLX2 | 0.057005 | 2 | let-7f-2-3p, miR-551b-5p |
| ELL | 0.057005 | 2 | miR-541-3p, miR-7-2-3p |
| EMB | 0.057005 | 2 | miR-646, miR-7-2-3p |
| GALNT2 | 0.057005 | 2 | miR-550a-5p, miR-485-5p |
| GLCE | 0.057005 | 2 | miR-485-5p, miR-519a-3p |

| | | | |
|---|---|---|---|
| GSG1 | 0.057005 | 2 | miR-511-5p, miR-646 |
| PDPN | 0.057005 | 2 | miR-489-3p, miR-485-5p |
| RND2 | 0.057005 | 2 | miR-15b-3p, miR-431-5p |
| TPT1 | 0.057005 | 2 | miR-1304-5p, miR-100-3p |
| VOPP1 | 0.057005 | 2 | miR-431-5p, miR-646 |
| WIPI2 | 0.057005 | 2 | miR-646, miR-376b-3p |
| DGKB | 0.003547 | 2 | miR-551b-5p, miR-7-2-3p |
| DGKI | 0.003547 | 2 | miR-127-5p, miR-7-2-3p |
| PCLO | 0.003547 | 2 | miR-127-5p, miR-7-2-3p |
| USP34 | 0.003547 | 2 | miR-127-5p, miR-485-5p |
| AFAP1 | 0.034386 | 2 | miR-646, let-7f-2-3p |
| CCDC83 | 0.034386 | 2 | miR-511-5p, miR-646 |
| DECR1 | 0.034386 | 2 | miR-511-5p, miR-646 |
| HEXIM1 | 0.034386 | 2 | miR-485-5p, miR-7-2-3p |
| IHH | 0.034386 | 2 | miR-431-5p, miR-326 |
| PROX2 | 0.034386 | 2 | miR-511-5p, miR-431-5p |
| SLC35G2 | 0.034386 | 2 | miR-376b-3p, miR-449b-5p |
| TIMP1 | 0.034386 | 2 | miR-519a-3p, miR-377-3p |
| C10orf10 | 0.028760 | 2 | miR-483-3p, miR-361-3p |
| GSE1 | 0.028760 | 2 | miR-361-3p, miR-489-3p |
| MAMLD1 | 0.028760 | 2 | miR-200b-3p, miR-7-2-3p |
| NOB1 | 0.028760 | 2 | miR-326, miR-646 |
| VAC14 | 0.028760 | 2 | miR-200b-3p, miR-377-3p |
| PPM1B | 0.002555 | 2 | miR-7-2-3p, miR-551b-5p |
| BBS4 | 0.001718 | 2 | miR-377-3p, miR-127-5p |
| HSPA12B | 0.016576 | 2 | miR-377-3p, miR-550a-5p |
| ZNF330 | 0.016576 | 2 | miR-377-3p, miR-483-3p |
| IL7 | 0.008980 | 2 | miR-376b-3p, miR-431-5p |
| PARM1 | 0.008980 | 2 | miR-377-3p, miR-1324 |
