# Supplementary material for: Adipose-Derived Mesenchymal Stromal Cells Treated with Interleukin 1 Beta Produced Chondro-Protective Vesicles Able to Fast Penetrate in Cartilage
Source: Cells. 2021 May 12;10(5):1180. doi: 10.3390/cells10051180 (PMC8151616; doi:10.3390/cells10051180)
Supplement: Supplementary file 1 [file cells-10-01180-s001.zip › Table S4.pdf]

Table S4: target genes of miRNAs down-regulated in ASCs treated with IL-1β.

Gene Symbol	p-value	Interactions	miRNAs
DNAJC21	0.000663	2	miR-656-3p, miR-500a-5p
SIK3	0.000570	2	miR-500a-5p, miR-656-3p
GRIN2B	0.002854	2	miR-500a-5p, miR-1265
NRIP1	0.001499	2	miR-656-3p, miR-1265
PLEKHG3	0.002953	2	miR-500a-5p, miR-1265
NR2C2	0.003913	2	miR-656-3p, miR-500a-5p
TTLL12	0.008525	2	miR-1265, miR-500a-5p
REL	0.019098	2	miR-500a-5p, miR-656-3p
