# Supplementary material for: Adipose-Derived Mesenchymal Stromal Cells Treated with Interleukin 1 Beta Produced Chondro-Protective Vesicles Able to Fast Penetrate in Cartilage
Source: Cells. 2021 May 12;10(5):1180. doi: 10.3390/cells10051180 (PMC8151616; doi:10.3390/cells10051180)
Supplement: Supplementary file 1 [file cells-10-01180-s001.zip › Table S5.pdf]

Table S5: target genes of miRNAs silenced in ASCs treated with IL-1β.

Gene Symbol	p-value	Interactions	miRNAs
TXNIP	0.012786	5	miR-378a-3p, miR-302d-3p, miR-135b-5p, miR-623, miR-23b-3p
LINC00598	0.042063	4	miR-142-3p, miR-130b-5p, miR-302d-3p, let-7e-3p
SRCAP	0.009764	4	miR-130b-5p, miR-194-5p, miR-623, miR-302d-3p
ZNF682	0.001530	4	miR-302d-3p, miR-122-3p, miR-23b-3p, miR-142-3p
ARL10	0.050683	3	miR-142-3p, miR-302d-3p, miR-122-3p
HMGA2	0.045980	3	miR-142-3p, miR-23b-3p, miR-194-5p
LPP	0.045068	3	miR-142-3p, miR-223-5p, miR-130b-5p
KCNK6	0.038112	3	miR-130b-5p, miR-302d-3p, miR-623
SPRED1	0.038112	3	miR-302d-3p, miR-194-5p, miR-589-3p
AGO1	0.035662	3	miR-628-3p, miR-378a-3p, miR-194-5p
CEP104	0.035662	3	miR-23b-3p, miR-223-5p, miR-302d-3p
HSPA1B	0.034865	3	miR-130b-5p, miR-378a-3p, miR-142-3p
IFNAR2	0.031773	3	miR-142-3p, miR-302d-3p, miR-566
KANSL1	0.031024	3	miR-23b-3p, miR-130b-5p, miR-623
FOXC1	0.029555	3	miR-378a-3p, miR-623, miR-223-5p
SLC7A11	0.026054	3	miR-142-3p, miR-223-5p, miR-302d-3p
KCNJ6	0.019205	3	miR-378a-3p, miR-130b-5p, miR-122-3p
HMGB2	0.018641	3	miR-23b-3p, miR-223-5p, miR-135b-5p
SORCS2	0.018086	3	miR-23b-3p, miR-302d-3p, miR-623
ICA1L	0.015961	3	miR-589-3p, miR-302d-3p, miR-566
INTS7	0.010894	3	miR-566, miR-142-3p, miR-589-3p
EMC1	0.010093	3	miR-130b-5p, miR-23b-3p, miR-302d-3p
FOXO1	0.008958	3	miR-132-5p, miR-130b-5p, miR-135b-5p
ACBD5	0.007903	3	miR-223-5p, miR-142-3p, miR-122-3p
RBM27	0.006618	3	miR-142-3p, miR-623, miR-130b-5p
EP300	0.004693	3	miR-194-5p, miR-130b-5p, miR-23b-3p
NLGN4X	0.004693	3	miR-223-5p, miR-23b-3p, miR-130b-5p
RGS5	0.003557	3	miR-142-3p, miR-223-5p, miR-23b-3p

ADAT2	0.003353	3	miR-623, miR-302d-3p, miR-223-5p
SCYL3	0.002967	3	miR-130b-5p, miR-135b-5p, miR-142-3p
GPS1	0.002280	3	let-7e-3p, miR-566, miR-132-5p
MARCKS	0.002126	3	miR-142-3p, miR-23b-3p, miR-135b-5p
RUNX2	0.000689	3	miR-135b-5p, miR-23b-3p, miR-628-3p
MC2R	0.055390	2	miR-23b-3p, miR-130b-5p
PALM2-AKAP2	0.055390	2	miR-223-5p, miR-130b-5p
RRAGD	0.055390	2	miR-23b-3p, miR-566
C11orf74	0.053717	2	miR-142-3p, miR-623
SPTY2D1	0.053717	2	miR-23b-3p, miR-194-5p
TMTC3	0.052063	2	miR-142-3p, miR-223-5p
TNIP3	0.052063	2	miR-142-3p, miR-302d-3p
FAAP24	0.050428	2	miR-130b-5p, miR-302d-3p
PSD3	0.050428	2	miR-130b-5p, miR-302d-3p
ATP2A2	0.048813	2	miR-142-3p, miR-223-5p
IKZF2	0.048813	2	miR-130b-5p, miR-623
NOTCH2	0.048813	2	miR-130b-5p, miR-23b-3p
TMED7	0.048813	2	miR-23b-3p, miR-142-3p
ZNF529	0.047217	2	miR-142-3p, miR-223-5p
HIPK3	0.045642	2	miR-187-3p, miR-378a-3p
MTPN	0.045642	2	miR-130b-5p, miR-589-3p
ZFX	0.045642	2	miR-142-3p, miR-130b-5p
CPSF7	0.044088	2	miR-378a-3p, miR-23b-3p
FNIP1	0.044088	2	miR-223-5p, miR-23b-3p
HCFC2	0.044088	2	miR-623, miR-130b-5p
STARD7	0.044088	2	miR-378a-3p, miR-23b-3p
KIAA1210	0.042554	2	miR-23b-3p, miR-194-5p
PLEKHA6	0.042554	2	miR-223-5p, miR-130b-5p
SNX5	0.042554	2	miR-132-5p, miR-302d-3p
TGFBR1	0.042554	2	miR-142-3p, miR-135b-5p

KCND3	0.041042	2	miR-142-3p, miR-302d-3p
RAB42	0.041042	2	miR-589-3p, miR-302d-3p
POU2F2	0.039551	2	miR-623, miR-130b-5p
EIF2S1	0.038082	2	miR-302d-3p, miR-130b-5p
SERF1B	0.038082	2	miR-302d-3p, miR-566
TAB2	0.038082	2	miR-23b-3p, miR-142-3p
B4GALNT3	0.036635	2	miR-378a-3p, miR-623
RNF38	0.036635	2	miR-142-3p, miR-23b-3p
STXBP2	0.036635	2	miR-130b-5p, miR-566
GALNT3	0.035210	2	miR-223-5p, miR-302d-3p
HNRNPR	0.035210	2	miR-130b-5p, miR-302d-3p
PRKACB	0.035210	2	miR-23b-3p, miR-302d-3p
USP37	0.035210	2	miR-142-3p, miR-130b-5p
NOTCH1	0.032430	2	miR-23b-3p, miR-623
RPS19	0.032430	2	miR-142-3p, miR-566
SLC22A23	0.032430	2	miR-378a-3p, miR-302d-3p
DPP8	0.031074	2	miR-135b-5p, miR-302d-3p
MBD6	0.031074	2	miR-142-3p, miR-223-5p
SSH2	0.031074	2	miR-130b-5p, miR-623
ZKSCAN4	0.029742	2	miR-623, miR-223-5p
AZF1	0.028435	2	miR-142-3p, miR-135b-5p
DCTN4	0.028435	2	miR-142-3p, miR-378a-3p
SEMA6D	0.028435	2	miR-23b-3p, miR-589-3p
ITGA1	0.027151	2	miR-223-5p, miR-130b-5p
DUSP10	0.025893	2	miR-623, miR-130b-5p
LRP6	0.023451	2	miR-628-3p, miR-130b-5p
SOX5	0.023451	2	miR-194-5p, miR-223-5p
FZD2	0.022268	2	miR-566, miR-130b-5p
PPARGC1B	0.022268	2	miR-23b-3p, miR-378a-3p
FAM222B	0.021112	2	miR-142-3p, miR-23b-3p

AKR1B10	0.019982	2	miR-628-3p, miR-142-3p
OXSR1	0.019982	2	miR-378a-3p, miR-130b-5p
FAM177A1	0.018879	2	miR-142-3p, miR-223-5p
BRWD3	0.017803	2	miR-378a-3p, miR-223-5p
CCDC93	0.017803	2	miR-623, miR-130b-5p
INSIG2	0.017803	2	miR-142-3p, miR-223-5p
TIMM17A	0.017803	2	miR-302d-3p, miR-130b-5p
COPA	0.016754	2	miR-23b-3p, miR-142-3p
COX6C	0.016754	2	miR-302d-3p, miR-628-3p
MMGT1	0.016754	2	miR-142-3p, miR-23b-3p
KLHL8	0.015734	2	miR-302d-3p, miR-628-3p
STAM	0.015734	2	miR-142-3p, miR-130b-5p
MYRF	0.014741	2	miR-623, miR-378a-3p
NOTCH2NL	0.013777	2	miR-122-3p, miR-194-5p
PXDN	0.012842	2	miR-589-3p, miR-623
CHD1	0.011937	2	miR-130b-5p, miR-194-5p
KBTBD2	0.011937	2	miR-142-3p, miR-302d-3p
MID1	0.011937	2	miR-130b-5p, miR-135b-5p
CLTA	0.011061	2	miR-23b-3p, miR-142-3p
FGF1	0.011061	2	miR-130b-5p, miR-589-3p
PCMTD2	0.011061	2	miR-142-3p, miR-23b-3p
USP5	0.011061	2	miR-223-5p, miR-23b-3p
MBNL2	0.010215	2	miR-302d-3p, miR-223-5p
FLNA	0.009400	2	miR-378a-3p, miR-23b-3p
NDFIP2	0.009400	2	miR-378a-3p, miR-223-5p
NSFL1C	0.009400	2	miR-142-3p, miR-130b-5p
TNFAIP8	0.009400	2	miR-23b-3p, miR-223-5p
ZNF273	0.009400	2	miR-628-3p, miR-23b-3p
SOCS6	0.008616	2	miR-23b-3p, miR-142-3p
TCP1	0.007863	2	miR-378a-3p, miR-23b-3p

UGT2B28	0.007141	2	miR-623, miR-130b-5p
APC	0.005795	2	miR-135b-5p, miR-142-3p
BCLAF1	0.005171	2	miR-142-3p, miR-194-5p
PLP1	0.005171	2	miR-223-5p, miR-130b-5p
S100A7A	0.004023	2	miR-23b-3p, miR-130b-5p
ZNF283	0.004023	2	miR-130b-5p, miR-122-3p
PROM1	0.003012	2	miR-142-3p, miR-130b-5p
ZNF473	0.002558	2	miR-378a-3p, miR-142-3p
SAMD1	0.001758	2	miR-623, miR-132-5p
SECISBP2	0.001412	2	miR-142-3p, miR-130b-5p
ECH1	0.000830	2	miR-378a-3p, miR-23b-3p
