# Supplementary material for: Adipose-Derived Mesenchymal Stromal Cells Treated with Interleukin 1 Beta Produced Chondro-Protective Vesicles Able to Fast Penetrate in Cartilage
Source: Cells. 2021 May 12;10(5):1180. doi: 10.3390/cells10051180 (PMC8151616; doi:10.3390/cells10051180)
Supplement: Supplementary file 1 [file cells-10-01180-s001.zip › Table S6.pdf]

Table S6: fitting parameters related to the EVs average depth, occupied area and occupied volume are listed. The Eq. (1) corresponds to the double exponential function used to fit the experimental data, while the Eq. (2) corresponds to the single exponential function. For each fitting equation are showed the computed parameters and the Sum of the Squared Errors (SSE) and the coefficient of determination (R^2).

		**Eq. (1)** $\boldsymbol{a\left(1-e^{-\frac{t}{\tau_a}}\right)+b\left(1-e^{-\frac{t}{\tau_b}}\right)}$ **Eq. (2)** $\boldsymbol{a\left(1-e^{-\frac{t}{\tau_a}}\right)}$					
		**Fitting parameters**				**Goodness of Fit**	
		$\boldsymbol{a}$	$\boldsymbol{\tau_a}$	$\boldsymbol{b}$	$\boldsymbol{\tau_b}$	**SSE**	$\mathbf{R^2}$
**Average Depth**	Eq. (1)	10.931 [μm]	34657 [s]	7.931 [μm]	2354.76 [s]	15.9566	0.991
	Eq. (2)	16.389 [μm]	12297 [s]	-		103.9954	0.862
**AverageArea**	Eq. (1)	18438 [$μm^2$]	21182 [s]	22102 [$μm^2$]	1956.24 [s]	2.35e+11	0.994
	Eq. (2)	38646 [$μm^2$]	8640 [s]	-		2.44e+11	0.995
**AverageVolume**	Eq. (1)	640955 [$μm^3$]	35935 [s]	148700 [$μm^3$]	2603.9 [s]	3.88e+10	0.994
	Eq. (2)	692731 [$μm^3$]	20948 [s]	-		7.26e+10	0.997
